# Antioxidants Acteoside and Orientin as Emerging Agents in Synergistic Cancer Therapy: A Focus on Innovative Applications

**DOI:** 10.3390/antiox14070855

**Published:** 2025-07-12

**Authors:** Jagoda Szkudlarek, Ludwika Piwowarczyk, Anna Jelińska

**Affiliations:** 1 Chair and Department of Pharmaceutical Chemistry, Poznan University of Medical Sciences, 3 Rokietnicka, 60-806 Poznań, Poland; ajelinsk@ump.edu.pl; 2 Doctoral School, Poznan University of Medical Sciences, 70 Bukowska, 60-812 Poznań, Poland

**Keywords:** acteoside, verbascoside, orientin, cancer, antioxidants, polyphenols, antitumor cytotoxic, lipid-based drug delivery systems (LBDDSs)

## Abstract

Cancers, particularly those resistant to treatment, stand as one of the most significant challenges in medicine. Frequently, available therapies need to be improved, underscoring the necessity for innovative treatment modalities. Over the years, there has been a resurgence of interest in natural plant substances, which have been traditionally overlooked as anticancer agents. A prime example of this is natural antioxidants, such as acteoside (ACT) and orientin (ORI), which offer novel approaches to cancer treatment, emphasizing liver cancer compared to other cancer types. They reduce oxidative stress by activating the Nrf2/ARE pathway and exhibit anticancer activity, e.g., decreasing Bcl-2 and Bcl-XL expression and increasing Bax levels. This review explores the individual effects of ACT and ORI and their synergistic interactions with sorafenib, temozolomide, 5-fluorouracil (for ACT), celecoxib, and curcumin (for ORI), highlighting their enhanced anticancer efficacy. In addition, ACT and ORI successfully integrate into various drug delivery systems (DDSs), including metal-containing carriers such as nanoparticles (NPs), nanoshells (NSs), quantum dots (QDs), and liposomes as representative examples of lipid-based drug delivery systems (LBDDSs). Advanced methods, including nanotechnology, offer potential solutions to low bioavailability, paving the way for the use of these substances in anticancer therapy.

## 1. Introduction

Cancer, a significant public health challenge on a global scale, ranks among the top global causes of death due to insufficient early-stage diagnostic tools [1]. In 2024, an estimated 2,001,140 people in the United States will receive a cancer diagnosis, with an assessed 611,720 of them succumbing to the disease [2], underscoring the critical need for ongoing cancer research and treatment advancements. During the 1980s, the pharmaceutical industry shifted its focus to developing targeted agents from synthetic compound libraries [3]. This led to a subsequent prioritization of targeted therapies that utilize antibodies or synthetic molecules over natural products by the 1990s. However, this change in focus was not permanent, and the result was a return to natural products, which played a crucial role in developing anticancer drugs [4]. Natural products, which boast intricate and complex structures shaped by evolution, have once again garnered attention as a valuable source for successful treatments, including in oncology [3].

Furthermore, numerous phytochemicals, including polyphenols (such as ACT and ORI), exhibit significant anticarcinogenic properties, preventing or slowing cancer progression by affecting cellular mechanisms [5]. ACT, also known as verbascoside or kusagin, is a phenylethanoid glycoside found in many dicotyledonous plants such as *Oleaceae*, *Bignoniaceae*, *Verbenaceae,* and *Labiatae* [6,7]. ACT has diverse effects, including hepatoprotective, neuroprotective, antiproliferative, and anticancer properties [8], with the antitumor effect dependent on the tumor’s location [9]. The second compound, ORI, luteolin-8-C-glucoside, is a water-soluble flavonoid C-glycoside that can be isolated from various medicinal plants such as *Ocimum sanctum*, *Commelina communis*, *Jatropha gossypifolia* and *Phyllostachys nigra* [10]. It is well known for its anti-inflammatory and anticancer properties [11].

Scientific research shows an advantage of ACT and ORI over other compound groups under investigation. Yücer et al. emphasized that ACT ranked among the top 10 compounds binding to anti-inflammatory proteins NLRP3 and NF-κB, and additionally, ACT is not associated with significant drug resistance mechanisms [12]. Vaziri-Amjad et al. highlighted, among others, ORI’s strong ability from a list of 79 herbal compounds to inhibit Janus-activated kinase 2 (JAK2), a key protein involved in cell proliferation, apoptosis, and metastasis, suggesting it is a potential target for tumor treatment [13]. Both compounds are antioxidants that help modulate oxidative stress by activating key cellular defense mechanisms. Studies have demonstrated that ACT and ORI reduce oxidative stress by activating the Nrf2 (nuclear factor erythroid 2-related factor 2)/antioxidant response element (ARE) signaling pathway [14,15,16].

However, despite many beneficial properties, polyphenols present challenges with low bioavailability due to interactions with the food matrix, liver-mediated metabolic processes, intestines, and microbiota [17]. For instance, ACT was rapidly absorbed and eliminated after a single oral dose, peaking at 30 to 45 min (T_max_) with a half-life of roughly 90 min. Its absolute bioavailability was approximately 4% [18]. In another study, the bioavailability of oral ACT was even lower and amounted to 0.12% [19]. ACT’s low bioavailability results from its degradation during gastrointestinal digestion, poor absorption, and efflux by P-glycoprotein (P-gp) [20]. However, for the second compound, ORI was not detectable in plasma after oral administration, but it was rapidly distributed to tissues and eliminated from plasma within 90 min following intravenous injection [21]. In another study, using oral raw bamboo extract in rats provided insights into C-glucosides’ bioavailability (ORI, homoorientin, vitexin, and isovitexin). They exhibited poor absorption, were undetectable in rat blood and urine, and over 50% were recovered within 12 h and excreted in feces within 24 h [22].

The solution to these problems may be nanotechnology and nanoparticles, which enhance phytochemicals’ bioavailability, solubility, stability, absorption, and circulation time while protecting them from premature bodily degradation [23]. Nanoscale materials are significant in delivering anticancer agents to tumor sites and offer a practical approach to cancer diagnosis and treatment [24]. Nanotechnology facilitates manipulating and engineering materials on a nanometer scale, giving rise to revolutionary advancements in cancer treatment [25]. Nanotechnology is extensively researched as a DDS to effectively target therapy with various oncological substances while minimizing drug exposure to healthy tissues [26]. Moreover, DDSs show significant improvements in the bioavailability of drugs compared to traditional methods. For instance, liposomal encapsulation increased the bioavailability of capsaicin by 3.34-fold [27]. Silica-coated flexible liposomes and curcumin-loaded flexible liposomes enhanced curcumin bioavailability by 7.76-fold and 2.35-fold, respectively, over curcumin suspensions [28]. Curcumin-loaded NPs showed a 9-fold increase in oral bioavailability compared to curcumin with piperine [29]. One of the subcategories of DDSs is LBDDSs. LBDDSs offer significant features such as safety, efficiency, biocompatibility, and versatility in delivery routes along with ease of formulation and scale-up [30,31]. LBBDSs hold promise for developing commercially viable pharmaceutical formulations suitable for topical, oral, pulmonary, or parenteral administration [30]. The review used the following databases to select publications: PubMed, Google Scholar, Web of Science (WoS), and Scopus. The keywords included “acteoside,” “verbascoside,” “orientin,” “cancer,” “cytotoxic,” “nanotechnology,” “nanoparticles,” “synergy,” “synergistic,” “acteoside with,” “verbascoside with,” and “orientin with.” This review compiles all available publications on a specific topic, filling a gap in the literature by providing the first comprehensive overview and compiling data for two selected natural compounds, ACT and ORI, in the cancer context.

## 2. Acteoside and Orientin Activity in Various Types of Cancer

**Figure 1:**
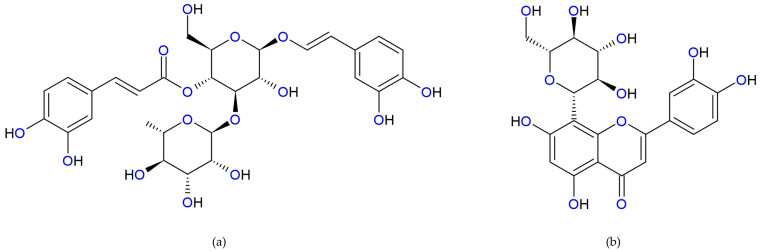
Chemical structures of (**a**) ACT and (**b**) ORI.

**Figure 2:**
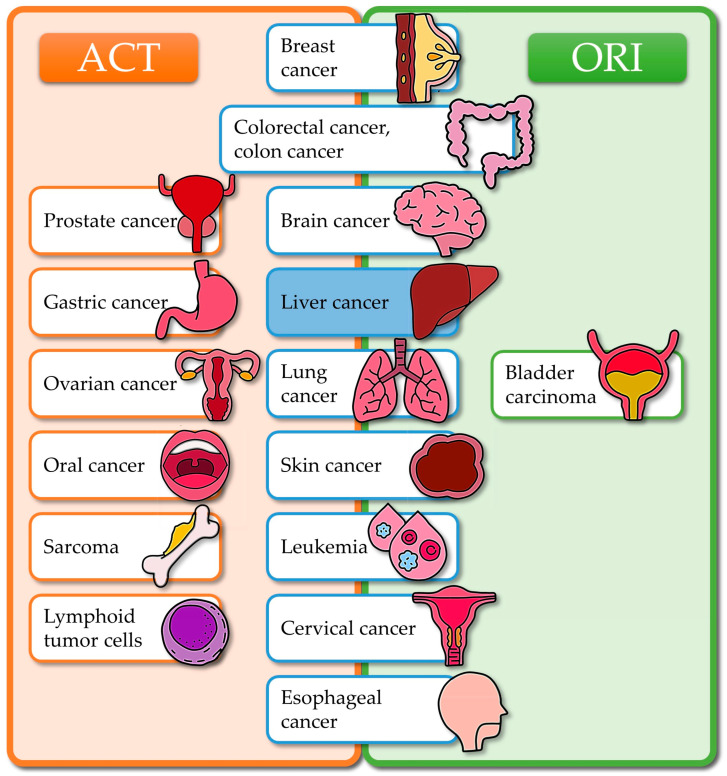
ACT and ORI studies were conducted on specific types of cancer.

Cancer is a major factor in global mortality rates, presenting a significant obstacle to increasing life expectancy worldwide [32]. Polyphenols like ACT and ORI have gained attention for their potential anticancer effects; their chemical structures are shown in Figure 1. Although not traditionally recognized as anticancer agents, ACT and ORI, both natural compounds, have recently demonstrated potential therapeutic effects in cancer treatment, as evidenced by recent publications. Figure 2 concludes the ACT and ORI studies on specific types of cancer.

### 2.1. Breast Cancer (BC)

Breast cancer (BC) is the most common cancer in developed nations and the second leading cause of cancer-related mortality among women [33]. The majority of BC fatalities result from distant metastasis [34], while breast cancer stem cells (BCSCs) drive tumor aggressiveness and pose a significant treatment challenge, which is mainly due to therapy resistance [35]. Current management approaches for BC typically involve surgical interventions and/or radiotherapy, which are frequently supported by adjuvant chemotherapy or hormone therapy [36]. The MCF-7 BC cell line has been extensively used in research by scientists for over half a century [37].

Asian diets, abundant in medicinal herbs, are linked to lower cancer rates, possibly due to antitumor properties. For instance, Liao et al. found that *Anisomeles indica* contains β-sitosterol, ovatodiolide, apigenin, and ACT, hindering the transcription of the MMP-9 gene through the NF-jB/AP-1 signaling pathway, thereby reducing invasiveness in MCF-7 [34]. Vasincu et al. demonstrated that ACT increased apoptosis rates in MCF-7 cells 2.5-fold compared to untreated cells. After 48 h of incubation at a concentration of 100 µg/mL, ACT decreased MCF-7 cell viability by 69.6% (IC_50_ values of 53.28 ± 7.87 µg/mL, 85.30 ± 12.6 µM) and triggered a 7.4% increase in the proportion of MCF-7 cells in the subG1 phase. Furthermore, ACT increased apoptosis rates in MCF-7 cells 2.5-fold compared to untreated cells [38]. In vitro, ACT shows cytotoxicity against MCF-7 cells with an IC_50_ of 134.83 μg/mL (215.86 µM), inducing apoptosis and modulating the Programmed Death-1 (PD-1) checkpoint and PD-L1 expression [39]. In a study on the effectiveness of ACT in inhibiting MCF-7 cells, Delazar et al. found that ACT from *Scrophularia subaphylla* L. exhibited IC_50_ values of 0.39 ± 0.015 µg/mL (0.624 ± 0.024 µM) [40]. Similar results were observed in studies by other scientists, where ACT from *Plantago lanceolata* L. demonstrated an IC_50_ of 219.1 ± 1.40 µM (after 24 h), 154.2 ± 2.71 µM (after 48 h), and 113.1 ± 2.81 µM (after 72 h) [41]. In another study, the IC_50_ values of ACT from *Phlomis nissolii* against MCF-7 cells were 0.127 μM at 24 h, 0.2174 μM at 48 h, and 0.2828 μM at 72 h. For another cell line commonly used in cancer research, MDA-MB-231 cells, derived from human breast adenocarcinoma, the IC_50_ values were 0.1597 μM at 24 h, 0.2584 μM at 48 h, and 0.2563 μM at 72 h [42]. In a separate study of the MDA-MB-231 cell line, the cytotoxic activity of ACT from *Plantago lanceolata* L. was characterized by an IC_50_ value of 312.2 ± 3.28 µM (after 24 h), 244.9 ± 4.96 µM (after 48 h), and 200.2 ± 2.45 µM (after 72 h) [41]. Interestingly, Daneshforouz et al. found that ACT, safe for normal HEK293T cells, reduced the viability of 4 T1 cells (mouse mammary tumor cells) dose-dependently with an IC_50_ of 116.7 μM. At 100, 117, and 130 μM concentrations, ACT increased caspase-3 and Bax expression and decreased Bcl-2 expression. At 130 μM, ACT upregulated Toll-like receptor 4 (TLR4) and myeloid differentiation primary response 88 (MyD88), while it downregulated NF-κB mRNA. Apoptosis in 4 T1 cells was induced at 117 μM ACT [43].

The effect of ORI on BC cells was also examined. Schuster’s research group confirmed the cytotoxic effects of ORI from *Cajanus cajan* on the MCF-7 cell line. The study reported an IC_50_ value for ORI exceeding 80 µg/mL (>178.42 µM) [44]. Furthermore, ORI from the aqueous ethanol extract of *Gleditsia triacanthos* L. leaves induced a 40.2% rate of cell death in MCF7 cells while showing no cytotoxic activity against liver or colon cell lines [45]. Interestingly, 12-O-tetradecanoylphorbol-13-acetate (TPA) induces the invasion of BC cells and elevates the expression levels of metalloproteinases (MMP-2, MMP-9) and interleukin-8 (IL-8). Conversely, ORI inhibits cell migration and invasion by suppressing the expression of MMP-9 and IL-8. Kim et al. demonstrated that ORI effectively impedes the migration and invasion of TPA-stimulated MCF-7 cells through the PKCα/ERK/AP-1/STAT3-mediated signaling pathways [46]. ORI was a component in other compounds and could have a crucial impact on the research results. Zu et al. identified ORI, vitexin, isovitexin, pinostrobin, and the stilbene cajaninstilbene acid in supercritical fluid extraction (SFE-CO_2_) extracts from *Cajanus cajan* (L.) Huth. The IC_50_ values of the SFE-CO_2_ extracts for MCF-7 cells were determined to be 0.0557 mg/mL (89.18 µM) [47]. In another study, Czemplik et al. found that vitexin, ORI, and isoorientin, abundant in flax straw, demonstrate cytotoxicity against MCF-7 cells, significantly inhibiting cell proliferation. Additionally, the induction of apoptosis was associated with alterations in the mRNA expression of pro-apoptotic genes: upregulation of Bax, caspase-7, -8, and -9, and downregulation of Bcl-2 mRNA expression, with no significant changes in p53 and mdm2 mRNA levels [48].

### 2.2. Colorectal Cancer, Colon Cancer

Colon cancer (CC), commonly referred to as colorectal cancer (CRC), is universally recognized in both research and clinical practice as a single tumor entity termed CRC [49]. While colonoscopy is the most effective way to prevent CRC, it may miss early molecular changes linked to tumors and dysplasia [50]. Chemotherapy, mainly 5-fluorouracil (5-FU), is the mainstay of CRC treatment, but 5-FU resistance (congenital or acquired) is the primary cause of treatment failure and relapse [51].

Fırat et al. found that ACT induced changes in the Bax/Bcl-2 ratio within COLO-741 CD133+ and CD133- cells and HTC-116 CD133+ and CD133- cells. This led to an elevation in caspase 3 activation, facilitating the initiation of the apoptotic process in these cells. Furthermore, ACT reduced the expression of stemness markers CD113 and Oct-4 in COLO-741 and HCT-116 CC cells [52]. In a different study, Zhou et al., in vivo, demonstrated that ACT at doses of 20, 40, and 80 mg/mL reduced the weight of CRC xenograft tumors by 42.79%, 53.90%, and 60.99%, respectively. Increased levels of HIPK2, p53, and Bax and decreased levels of Bcl-2 accompanied this reduction. Additionally, the apoptosis rates for HCT-116 cells treated with 25, 50, and 100 μM ACT were 10.83 ± 1.28%, 11.25 ± 1.54%, and 20.19 ± 2.87%, and for HT-29 cells, the apoptosis rates were 18.92 ± 6.12%, 21.57 ± 4.05%, and 25.14 ± 6.73%. The IC_50_ values for ACT at 24, 48, and 72 h were 208.89, 97.86, and 63.51 μmol/L (208.89, 97.86, and 63.51 µM) for HCT-116; 144.5, 108.82, and 66.68 μmol/L (144.5, 108.82, and 66.68 µM) for HT-29; 83.83, 59.62, and 43.96 μmol/L (83.83, 59.62, and 43.96 µM) for LoVo; and 52.73, 42.42, and 29.05 μmol/L for SW620 (52.73, 42.42, and 29.05 µM) [53]. The ACT from *L. brevituba* against HCT-116 cancer cells exhibited an IC_50_ value greater than 100 μM [54]. Interestingly, incubating HT-29 cells with 100 µg/mL of ACT for 48 h resulted in a 65.6% decrease in cell viability (IC_50_ values at 75.20 ± 4.11 µg/mL, 120.40 ± 6.58 µM) and a 5.1% increase in the proportion of cells in the subG1 phase. ACT enhanced apoptosis rates in HT-29 cells by 7.5-fold compared to control cells, and it raised reactive oxygen species (ROS) levels by 4.4%, 7.1%, 13.2%, and 19.6% after 1, 2, 3, and 24 h of incubation in tumor cells [38]. However, the IC_50_ value of ACT from *Scrophularia subaphylla* L. for the same cell line (HT-29) was measured at 0.93 ± 0.06 µg/mL (1.489 ± 0.096 µM) [40]. ACT demonstrated anti-invasion and anti-metastatic effects in HT29 cells by suppressing the Rac-1, HIF-1α, and zinc finger E-box binding homeobox 1 (Zeb-1) signaling pathway [55]. In another study, the ACT from *Plantago lanceolata* L. showed IC_50_ values of 507.6 ± 4.05 µM (24 h), 466.9 ± 8.71 µM (48 h), and 280.3 ± 6.13 µM (72 h) for the Caco-2 cell line [41]. Additionally, ACT exhibited an IC_50_ value of 73.6 ± 4.9 μM for SNU-C5 human colon cancer cells [56].

Studies also confirm the anticancer activity of ORI against CC and CRC. Schuster et al. reported that *Cajanus cajan* and its active compounds, including ORI, demonstrated an IC_50_ value exceeding 80 µg/mL (>178.42 µM) on the cell line CaCo-2 [44]. ORI inhibits Caco-2 cells in vitro with an IC_50_ value of 85.58 µg/mL (190.86 µM) [57]. Interestingly, Fayed et al. explored interactions between 32 phytochemical compounds from *Heliotropium ramosissimum* and CC antigen 10 binding sites. ORI showed affinity, with low binding energy values at −13.5655 kcal/mol (RMSD = 1.16 Å), comparable to doxorubicin (−14.0088 kcal/mol, RMSD = 1.71 Å). ORI establishes interactions with Ser73AA through hydrogen bonding and engages in π–H interaction with Glu76 [58]. Another research study demonstrated the potential of ORI in inhibiting HT29 cell proliferation by cellular morphology alterations, chromatin condensation, membrane blebbing, and nuclear fragmentation. ORI induced G_0_/G_1_ cell cycle arrest by regulating cyclins and cyclin-dependent protein kinases, impeding cell cycle progression into the S phase. ORI reduced the expression of Bcl-2 and Bcl-XL while increasing the levels of Bax and Bid. Additionally, ORI treatment led to an elevated expression of the tumor suppressor p53 [59]. An investigation on Wistar rats demonstrated ORI’s anticancer effects against colonic aberrant crypt foci (ACF) induced by 1,2 dimethylhydrazine (DMH). ORI effectively hinders the progression of colonic ACF and impedes the transformation of preneoplastic lesions into malignant neoplasms in CRC. ORI plays a regulatory role in antioxidant defense by intervening in the metabolic activation of DMH by inhibiting phase I biotransformation enzymes and increasing phase II enzymes, which facilitates detoxification and enhances carcinogen excretion from the lumen [11]. Similarly, in male albino Wistar rats, ORI prevented and inhibited CC induced by DMH. Additionally, the increase in biotransforming bacterial enzymes induced by DMH was effectively suppressed by ORI [60]. Another study confirms ORI’s efficacy in reducing inflammation and proliferation in DMH-induced CRC rats. ORI statistically significantly improved tumor marker levels (CEA and CA19-9) and suppressed proliferation markers (PCNA and Ki67). ORI effectively reduced inflammatory mast cells, decreased NF-κB and cytokine (TNF-α and IL-6) expression, and downregulated inflammatory inducible enzyme (iNOS and COX-2) overexpression [61].

### 2.3. Brain Cancer

Glioblastoma multiforme (GBM) is the most malignant and invasive glioma of the central nervous system (CNS) [62]. Over the past three decades, the five-year survival rate for patients with GBM has remained at around 4–5% [63]. Standard treatments (surgery, radiotherapy, chemotherapy) often prove ineffective with limited improvement in progression-free survival [64,65].

Hei et al. demonstrated in vitro (U87 and U251 cells) and in a U87-luciferase orthotopic xenograft mouse model that ACT inhibits GBM cell growth, reduces the receptor tyrosine kinase (c-Met) and epithelial-to-mesenchymal transition (EMT) marker levels (snail, vimentin, zeb1), and exerts antitumor effects by directly targeting c-Met, leading to its degradation via the ubiquitination–proteasome pathway [66]. Furthermore, ACT treatment inhibited the proliferation, migration, and invasion of U87 cells while enhancing apoptosis through the activation of SHP-1 and suppression of STAT3 phosphorylation. Jia et al. exhibit elevated expression of Src homology across two domain-containing protein tyrosine phosphatase 1 (SHP-1) and decreased the expression of the phosphorylated signal transducer and activator of transcription 3 (p-STAT3) in ACT-treated GBM compared to controls [67]. In another study, ACT suppressed HMGA2 (high mobility group A2) expression and inhibited the Wnt/β-catenin signaling pathway, reducing cell viability, invasion, and migration and impeding tumor growth in U87 and U251 cells [8]. Interestingly, the research conducted by Wang et al. demonstrated the inhibitory effect of ACT on U87MG, T98G, U118, and U251MG cells and a patient-derived GBM cell line. Researchers have confirmed that CD44 dimerization is vital for promoting GBM stem cell traits and drug resistance. ACT suppressed stem cell-like properties and tumor growth in vitro and in vivo, significantly extending survival in xenografted mice (U251MG) [68]. In addition, ACT treatment enhances miR-7-5p expression in U87 and U251 cells and transfers it to recipient GBM cells via exosomes. This decreased epidermal growth factor receptor (EGFR) levels by miR-7-5p, deactivating the phosphatidylinositol 3-kinase (PI3K)/protein kinase B (Akt) signaling pathway. Consequently, there was an inhibition of GBM cell proliferation, migration, invasion, and microtubule formation in vitro, along with a reduction in cancer formation and metastasis in vivo, as demonstrated by a model of nude mice injected with U87 cells [69]. Moreover, ACT from *Plantago lanceolata* L. exhibited an IC_50_ of 274.3 ± 2.61 µM (24 h), 201.9 ± 4.90 µM (48 h) and 156.6 ± 4.74 µM (72 h) against the U-138 MG cells [41]. However, another study reported lower IC_50_ values for ACT (in DMSO), with a value of 44.0 ± 4.1 µM in U-138 MG cells and 85.0 ± 4.3 µM in T98G cells [70]. ACT demonstrated limited effectiveness against the other glioblastoma cell line U251-MG with IC_50_ values of 3152.7 ± 4.15 µM (24 h), 2412.5 ± 7.97 µM (48 h), and 1165.3 ± 6.05 µM (72 h) [41].

Although less studied than ACT in GBM, ORI showed efficacy in inhibiting GBM cell viability. Pirvu et al. showed in vitro that ORI effectively inhibited the viability of U87 MG cells with an IC_50_ value of 30.91 µg/mL (68.94 µM) [57].

### 2.4. Liver Cancer

Liver cancer is highly prevalent amongst malignancies, with approximately 90% of primary liver cancers being hepatocellular carcinoma (HCC), which is currently the fifth most common cancer worldwide [71,72]. Chronic hepatitis B and C infections are responsible for 78% of HCC cases worldwide [73]. Sorafenib, regorafenib, and lenvatinib are the only established treatments for advanced HCC, providing weak overall survival benefits [74].

Continuous alcohol consumption triggers the translocation of NFκB into the nucleus by activating IκB kinase. Khullar and colleagues demonstrated that in HepG2 and Wistar rat models, ACT effectively mitigated liver damage caused by alcohol by restoring serum and tissue parameters and suppressing inflammation by inhibiting the NFκB/IκB signaling pathway [75]. In another study, Ma et al. showed the inhibitory effects of ACT on cell proliferation, colony formation, and migration in human HCC cell lines (BEL7404, HLF, JHH-7) and its impact on suppressing angiogenesis. Moreover, the continued treatment of JHH-7 cells with ACT led to an increase in p53 and a reduction in kallikrein-related peptidase (KLK1, KLK2, KLK4, KLK9, and KLK10) gene levels [7]. ACT from *Plantago lanceolata* L. demonstrated an IC_50_ of 261.3 ± 3.52 µM (24 h), 219.6 ± 2.65 µM (48 h), and 173.8 ± 1.59 µM (72 h) against HepG2 [41]. Xiang et al. reported an IC_50_ value greater than 100 μM for ACT from *L. brevituba* against HepG2 cells [54]. Lee et al. demonstrated a lower IC_50_ value of 53.1 ± 6.9 μM for HepG2 cell lines [56], while Saracoglu et al. demonstrated an IC_50_ value of 61.98 µg/mL (99.23 µM) for ACT on rat hepatoma dRLh-84 cells [76]. Schisandra chinensis lignans and ACT reduce CCL20 expression, inhibit hepatoma growth and migration, and enhance apoptosis by modulating the ERK1/2 pathway. In vivo, treated nude mice exhibited a notable decrease in tumor growth and size [77].

ORI is a promising anticancer agent in liver cancer primarily through its antiproliferative effects on HepG2 [78]. Tao et al. revealed that ORI hinders the proliferation and migration of the HepG2 and Huh7 cells, which is related to modulation in the expression of molecules associated with these processes in these cells. Notably, this inhibitory effect targets the NF-κB signaling pathway [79].

#### Acteoside and Orientin vs. Other Natural Antioxidants in Liver Cancer

Emerging evidence suggests that ACT and ORI may outperform well-known polyphenols such as curcumin, epigallocatechin gallate, and resveratrol in HCC. ACT significantly suppresses HCC progression by inhibiting proliferation, migration, and angiogenesis in BEL-7404, HLF, and JHH-7 cells through increased p53 activity, the downregulation of KLK expression, and the inhibition of angiogenesis [7]. Tao et al. further reported the modulation of MIF expression and regulation of MAPK phosphorylation, thereby promoting autophagy and apoptosis while simultaneously inhibiting cellular invasion and migration [80]. In contrast, curcumin and resveratrol often exhibit similar in vitro effects only when delivered through nanoformulations due to the poor stability of free compounds [81]. Molecular docking analyses reveal that ACT binds multiple oncogenic kinases (e.g., RAF1, BRAF, FGFRs, AXL) with affinities comparable to or better than curcumin or epigallocatechin gallate [72]. ORI inhibits NF-κB-driven proliferation and migration in Huh7 and HepG2 cells, similarly to curcumin, but displays lower cytotoxicity and more favorable pharmacokinetics [79]. In contrast, curcumin is subject to rapid hepatic metabolism, conjugation, and extremely low oral bioavailability, prompting the excessive use of delivery systems or prodrugs [82,83]. Resveratrol also exhibits limited bioavailability due to rapid metabolism and low stability [84]. Conversely, ORI demonstrates rapid liver accumulation, minimal tissue retention, and independence from CYP450 metabolism, suggesting more predictable clearance [79].

Meanwhile, both ACT and ORI show pronounced antioxidant and hepatoprotective effects, protecting against ROS and mitochondrial dysfunction in liver injury models, which is a combined profile rarely matched by curcumin/resveratrol in native form [79,84]. ACT also synergizes with oxaliplatin to improve antitumor efficacy and reduce hepatotoxicity via PI3K/Akt inhibition [80,85], whereas curcumin or resveratrol typically require nanoparticle delivery to achieve similar outcomes [81]. ACT and ORI exhibit superior intrinsic stability, multitarget anticancer action, favorable liver targeting, and safety compared to curcumin and resveratrol; however, head-to-head preclinical comparisons are still needed to substantiate these advantages.

### 2.5. Lung Cancer

Approximately 80% of lung cancer cases are non-small cell lung cancer (NSCLC), which has a higher 5-year survival rate (23%) than small cell lung cancer (SCLC) [86,87]. Although tobacco smoking is the prominent risk factor for lung cancer, survival also depends significantly on age, morphology, and stage [88,89]. Treatment includes surgery, radiation therapy, chemotherapy, targeted therapy, or a combination [90].

Vasincu et al. found that 100 µg/mL ACT reduced A549 cell viability by 60.9% after 48 h (IC_50_ values were 71.56 ± 5.33 µg/mL, 114.57 ± 8.53 µM) and increased the proportion of A549 cells in the subG1 phase by 9.9%. Additionally, ACT increased apoptosis rates in A549 cells 2.3 times compared to control cells and increased ROS levels by 9.5%, 21.2%, and 37.8% after 2, 3, and 24 h of incubation in tumor cells [38]. In another study, the ACT from the extract of *L. brevituba* against A549 cancer cells exhibited IC_50_ values of 93.92 ± 2.72 μM [54]. Other research has confirmed the anti-lung cancer potential of the ethanolic extract from *Clerodendrum chinense* leaves, which contains ACT, isoverbascoside, and hispidulin. The IC_50_ values for ACT against A549 were 248.40 ± 15.82 µg/mL (397.70 ± 25.33 µM), indicating its contribution to the extract’s antiproliferative effects. The extract exhibited a selectivity index (SI) of 2.4 and 2.8 after 24 and 48 h of incubation, respectively, compared to normal keratinocytes. At a concentration of 250 µg/mL, the extract resulted in a 21.67% induction of late-stage cell apoptosis, which is concomitant with an augmentation in the induction of ROS. The extract significantly inhibited lung cancer cell colony formation in a dose-dependent manner and cancer cell migration compared to the control group [90]. In another study, Lee et al. demonstrated that ACT had an IC_50_ value of 62.5 ± 4.6 μM against 3LL Lewis mouse lung carcinoma cells [56].

As mentioned above, ACT has a positive effect on A549 cells. Similarly, Khalil et al. demonstrated the strong effect of ORI on A549 cell lines, showcasing its potent inhibition of cell migration and invasion. ORI (25 μM) positively regulates COX-2, iNOS, and BCL-2 markers. Notably, ORI decreased COX-2 expression by destabilizing its mRNA, reducing functional availability for prostaglandin-2 release [91].

### 2.6. Skin Cancer

Melanoma is a skin cancer caused by abnormal melanocyte growth with risk factors including early-life sunburns, numerous nevi, and giant congenital or dysplastic nevi [92,93]. Early detection can yield a 95% 5-year survival rate for most skin cancers [94], but metastatic malignant melanoma has a lower rate of 5% to 19%, depending on metastasis extent and location [95]. Treatments include surgical excision, immunotherapy, gene therapy, and biochemotherapy [92].

In mouse studies, ACT treatment suppressed melanoma progression, reduced inflammation, ROS, and levels of CD31 (a marker for assessing angiogenesis), survivin (known for inhibiting apoptosis), Ras, Raf1, and p-STAT3, whereas increasing the expression of estrogen receptor (Erβ), Bax, cleaved caspase-3, and cleaved caspase-9 [96]. In another study, ACT has toxicity in B16.F1 and B16.F10 melanoma cells, increasing the phosphorylation of Creb1 in B16.F1 cells and Jun in B16.F10 cells. In the less ACT-sensitive B16.F10 line, ACT inhibited the phosphorylation of several proteins (p53, Stat3, Fak1, Gsk3A, Mp2k1, Ptn11, Rs6, Tf65, Wnk1). In a melanoma mouse model (B16.F1), intraperitoneal and oral ACT administration upregulated tumor antioxidant responses, but only the first suppressed tumor growth and activated antitumor immune responses [9]. Interestingly, Son et al. showed that ACT reduced tyrosinase activity and inhibited melanin production in B16.F10 cells by stimulating extracellular signal-regulated kinase (ERK signaling) and suppressing the expression of microphthalmia-associated transcription factor (MITF), tyrosinase, and tyrosinase-related protein-1 (TRP-1) [97]. The antiproliferative effect of ACT from *Clerodendron bungei* (leaves) and *Clerodendron trichotomum* (leaves, bark) against B16.F10 cells is confirmed by Nagao et al.’s study showing a GI_50_ of 8 mM [98]. Similarly, ACT from *L. dulcis* and *L. canescens* showed a GI_50_ of 11 mM [99].

However, ORI’s efficacy against skin cancer remains relatively understudied. However, it is crucial to highlight that ORI could be a potential antibiotic therapy for *S. aureus*-associated skin cancer. Actinic keratosis and squamous cell carcinoma (SCC) are commonly linked to an excess of multidrug-resistant *Staphylococcus aureus*. The secretion of virulence products by *S. aureus* induces chronic inflammation, leading to skin cancer, which is a process regulated by the staphylococcal accessory regulator (SarA). In a molecular docking study targeting the SarA protein of *S. aureus*, ORI exhibited significant binding energy of −8.6 kcal/mol [100].

### 2.7. Leukemia

Leukemias, characterized by dysfunctional immune cells and the disruption of typical bone marrow activity, include acute leukemia, which is a leading cause of cancer-related death [101,102]. Despite treatments like chemotherapy, radiotherapy, and immunotherapy, the 5-year survival rate remains low (30–50%) due to treatment toxicity and disease relapse [103]. The HL-60 cell line originated from a patient with acute promyelocytic leukemia and is a valuable in vitro model for studying leukemic and normal cell proliferation and differentiation [104].

The study by Inoue and colleagues proved that ACT induced cell death in HL-60 cells (IC_50_ of 26.7 µM) and prompted the characteristic internucleosomal breakdown of chromatin DNA associated with apoptosis [105]. However, Lee et al. showed that ACT impeded cell cycle progression in the G_1_ phase in HL-60 cells, which occurs earlier than the beginning of differentiation and seems associated with increased expression of the p21^CIP1/WAF1^ and p27^KIP1^ proteins. This effect is accompanied by reduced cyclin-dependent protein kinase (CDK) activities such as CDK2, CDK4, and CDK6. Moreover, the ACT study demonstrated a concentration- and time-dependent suppression of HL-60 cell proliferation with an IC_50_ value of 38.3 ± 1.3 μM [56]. Furthermore, ACT has been verified to exhibit anticancer properties against lymphocytic leukemia cells in P-388 mice with an effective dose (ED_50_) of 2.6 µg/mL (4.16 µM) [106].

Interestingly, Aragão et al. demonstrated that the methanol leaves extract from *Cecropia pachystachya* (CPM) inhibited the proliferation of more than 50% in human T-cell lymphoblast-like cells (Jurkat cells), HL60 cells, and HL60.Bcl-2 cells (which are resistant to anticancer drugs due to Bcl-2 expression). The study suggested that the identified ORI and isoorientin were responsible for CPM’s cytotoxic effects in these cell lines [107].

### 2.8. Cervical Cancer

Cervical cancer, the dominant cancer among women in developing nations, is primarily caused by human papillomavirus (HPV), and early detection by cytological examination and the treatment of precancerous lesions is vital for prevention [108,109,110]. The HeLa cell line, derived from Henrietta Lacks in 1951 without her consent [111], is crucial in cervical cancer research [112]. Interestingly, the Hep-2 line, initially described as laryngeal cancer cells, is contaminated with HeLa cells, highlighting issues of misidentification and cross-contamination [113].

Scientists confirm the cytotoxic and antiproliferative effects of ACT on HeLa cells. Saracoglu et al. demonstrated its cytotoxicity with an IC_50_ value over 200 μg/mL (>320.20 µM) [76]. ACT (from *L. dulcis* and *L. canescens*) had a GI_50_ value of 50 mM [99], while ACT (from leaves and bark of *Clerodendron bungei* and *Clerodendron trichotomum*) had a GI_50_ of 66 mM [98].

ORI exhibited potent inhibitory effects on HeLa cell proliferation in a dose-dependent manner. Alterations in pivotal regulatory proteins marked the apoptotic pathway stimulated by ORI. ORI grew Bax expression while reducing Bcl-2 expression, significantly increasing the Bax/Bcl-2 ratio. ORI also activated caspase-3 and caspase-9, which are key proteins in apoptotic pathways [114]. Additionally, *Cajanus cajan* and its active compounds, including ORI, demonstrated notable cytotoxic effects on the HeLa cell line with an IC_50_ value exceeding 80 µg/mL (>178.42 µM) [44].

### 2.9. Esophageal Cancer

Esophageal cancer (EC) is frequently diagnosed at an advanced stage due to the absence of early clinical symptoms [115], resulting in a 5-year survival rate of approximately 15–25% [116]. Histologically, EC is classified into two main categories: adenocarcinoma (EAC) and squamous cell carcinoma (ESCC) [117]. The now-established standard of care for EC is trimodal therapy, consisting of chemoradiation followed by surgical resection [118].

In ESCC, there is heightened activity of high mobility group box 1 (HMGB1) and receptor for advanced glycosylation end-products (RAGE). In vitro studies by Ji et al. exhibited that ACT has antitumor activity in ESCC by inhibiting the activation of cell division cycle 42 (CDC42) through the HMGB1/RAGE signaling [119].

However, An et al. demonstrated an inhibitory effect of ORI on the proliferation of EC-109 cells, with inhibition rates increasing with concentration and reaction time, a concomitant increase in p53 gene expression levels, and a decrease in bcl-2 gene expression [120].

### 2.10. Other Types of Cancer

ACT and ORI have demonstrated effectiveness against various cancer types beyond those mentioned earlier. Accessible studies indicate that ACT has been more extensively studied than ORI, showing effectiveness against prostate, gastric, ovarian, oral cancers, sarcoma, and lymphoid tumors. Meanwhile, ORI has demonstrated positive effects on human bladder carcinoma.

Mulani et al. found that ACT was more effective than other phenylethanoid glycosides, such as chinacoside, calceolarioside-A, and calceolarioside-B, in inhibiting prostate cancer cell proliferation [121]. ACT treatment reduced the proliferation, migration, and invasion capabilities of human prostate cancer cell lines, Du-145 and PC-3 cells, by suppressing the HMGB1/RAGE axis; there was a reduction in the progression of TGF-β-associated epithelial–mesenchymal transition (EMT). Additionally, ACT reduced the expression of EMT promoters, Snail and Slug, and enhanced E-cadherin expression while suppressing the TGF-β pathway [122]. Interestingly, Marcoccia et al. discovered that ACT from *Verbascum xanthophoeniceum* and extract from this plant do not have cytotoxic effects on human LNCaP prostate epithelial cells. In contrast, both the extract and ACT demonstrated an inhibitory effect on the secretion of dihydrotestosterone (DHT)-induced free and total prostate-specific antigen (PSA), indicating a discernible anti-androgenic activity [123].

ACT has demonstrated activity against human gastric cancer cells, including MGc80-3 and MK-1. Ji et al. conducted experiments on mice and found that 20 µmol/L of ACT treatment induces redifferentiation in MGc80-3 cells and reverses their malignant phenotype [124]. ACT from the extract of *L. brevituba* exhibited an IC_50_ value of 45.92 ± 4.48 μM against MGc80-3 cancer cells [54]. However, Abe et al. determined that ACT from *L. dulcis* and *L. canescens* demonstrated an antiproliferative effect with a GI_50_ value of 35 mM against MK-1 cells [99]. Similarly, Nagao et al. reported a comparable GI_50_ value of 40 mM for ACT from *Clerodendron bungei* leaves and ACT from *Clerodendron trichotomum* leaves and bark against MK-1 cells [98].

Research confirms ACT’s impact on ovarian cancer (OC). Budzianowska et al. showcased ACT’s (from *Plantago lanceolata* L.) cytotoxicity against OVCAR-3 cells with an IC_50_ value of 314.1 ± 3.52 µM (24 h), 232.0 ± 5.08 µM (48 h), and 162.8 ± 3.41 µM (72 h) [41]. Additionally, ACT raised the CD86+/CD206+ ratio (M1/M2 ratio macrophage), indicating a shift toward M1 polarization in macrophages, and enhanced the expression of IL-6 and CXCL10 (M1 biomarkers). The induction of M1 polarization by ACT effectively stopped the proliferation of OC cells (SKOV3 and A2780) [125].

A positive effect was also reported on oral cancer cell lines. In a xenograft oral squamous cell carcinoma (OSCC) mouse model study, Zhang et al. found that ACT reduced the viability and spread of human OSCC cell lines (HN4 and HN6) while stimulating apoptosis. Additionally, ACT effectively downregulated the mRNA and protein expression levels of matrix metalloproteinase-9 (MMP-9), thus impeding tumor cell metastasis [126]. ACT stopped the development of OSCC (SCC9 and UM1) by blocking the methyltransferase-3 (METTL3)-regulated microRNA (miR)–31-5p/homeodomain interacting protein kinase-2 (HIPK2) axis [127].

ACT shows promise as a potent anticancer drug for treating fibrosarcoma metastasis. Hwang et al. showed that ACT hinders the invasion and migration of human fibrosarcoma HT-1080 cells induced by phorbol-12-myristate-13-acetate (PMA) through the regulation of Ca^2+^-dependent calmodulin-dependent protein kinase, extracellular regulated kinase (ERK), and JNK/NF-κB signaling pathways [128]. Furthermore, Saracoglu et al. demonstrated ACT’s cytotoxicity against S-180 sarcoma cells with an IC_50_ of 29.6 µg/mL (47.39 µM). In the same study, researchers demonstrated the cytotoxic effect of ACT on P-388/D1 cells, a mouse lymphoid tumor cell line, with an IC_50_ of 221.0 µg/mL (353.83 µM) [76]. In another study, ACT exhibited an IC_50_ value of 42.5 ± 2.1 μM in U-937 human histiocytic lymphoma cells [56].

However, Tian et al. demonstrated that in vitro ORI positively affected T24 bladder carcinoma cells by inhibiting cell proliferation, inducing cell cycle arrest, diminishing cell viability, and stopping the expression of inflammatory mediators. Additionally, ORI enhanced cell apoptosis by inhibiting the Hedgehog signaling pathway and NF-κB [129].

The following tables outline the IC_50_ values of ACT (Table 1) and ORI (Table 2) across a spectrum of cancer types.

The variability in reported IC_50_ values for ACT and ORI likely results from biological differences (e.g., cell line genetics, transporter expression), variations in experimental conditions (e.g., incubation time, medium composition), and the choice of cytotoxicity assays with differing sensitivities. Technical factors such as compound purity, solvent effects, and storage conditions, as well as divergent data analysis methods and reporting standards, further contribute to these discrepancies. These observations highlight the importance of standardized protocols and reporting to improve the comparability of IC_50_ data across studies [130,131,132,133].

## 3. Acteoside and Orientin in Synergy in Cancer

**Figure 3:**
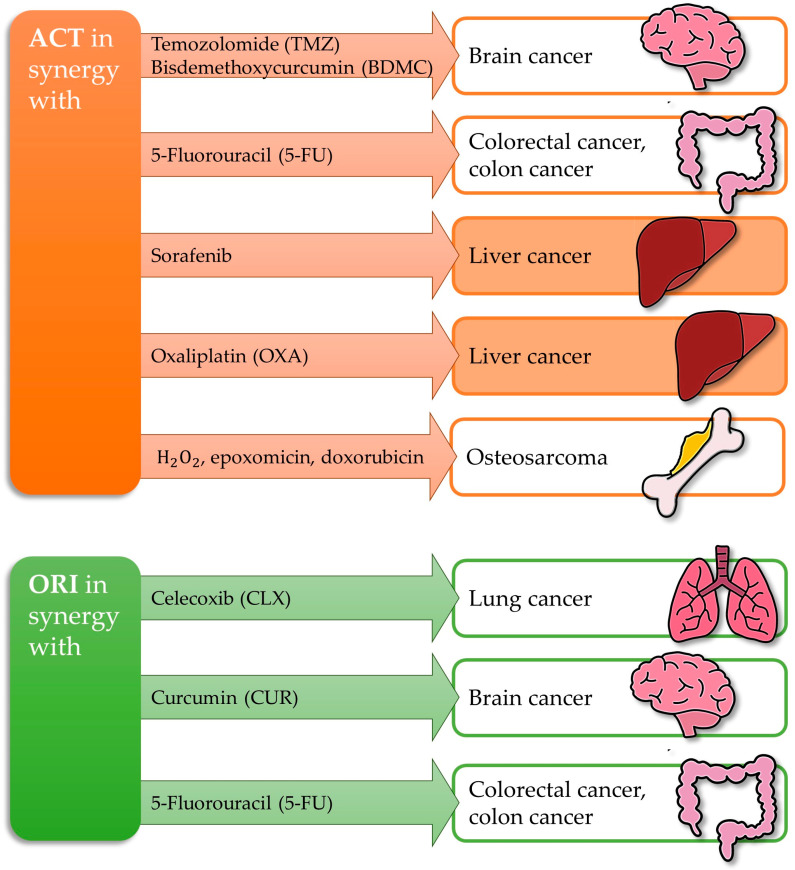
ACT and ORI in synergy in the treatment of cancer.

Synergy, a significant aspect of combined therapies, highlights the potential of natural phytochemical interactions, particularly the abundance of phenolic compounds in polyphenol-rich extracts, to enhance anticancer activity beyond that of individual compounds [134,135]. Promising results confirm the significant potential of combined therapy with ACT or ORI. Figure 3 summarizes the previous studies, demonstrating how ACT and ORI work in synergy in cancer treatment.

### 3.1. Acteoside in Synergy in Cancer

The available data suggest that combining ACT with other substances could improve cancer treatment, including GBM, where resistance to the primary chemotherapy temozolomide (TMZ) leads to treatment failure in around half of patients because they become resistant to TMZ [136]. Research using the C6 rat glioblastoma cell line suggests combining TMZ and ACT enhances therapeutic efficacy, significantly reducing cell viability and migration compared to TMZ or ACT alone [137]. Furthermore, bisdemethoxycurcumin (BDMC) has anticancer activity, specifically against the GBM 8401/luc2 cell xenograft model, inducing apoptosis and stopping tumor cell proliferation [138]. Piwowarczyk et al. demonstrated that the combination of ACT and BDMC resulted in IC_50_ values of >100 µM in T98G cells and 69.0 ± 5.2 µM in U-138 MG cells [70].

Another compound, 5-FU, is standard chemotherapy for CRC in palliative and adjuvant contexts, but low patient response rates underscore ongoing challenges in CRC treatment despite advancements [139]. Attia et al. exhibited a potential role for ACT as a complementary treatment to alleviate CRC cell resistance to 5-FU possibly by targeting the phosphoinositide 3-kinase (PI3K)/AKT signaling pathway. They identified a synergistic cytotoxic interaction between 5-FU and ACT, except for G_1_ cell cycle arrest, in Caco-2 and HCT-116 cells. Furthermore, an increase in apoptosis was observed mainly through the modulation of Bax and Bcl-2 and, to a lesser extent, Bcl-xL and p53 protein. The IC_50_ values for 5-FU and ACT were 1.199 µM and 1.088 µM in HCT-116 cells and 0.269 µM and 0.956 µM in Caco-2 cells, respectively. Interestingly, a combination of 5-FU with ACT (0.1 μM) produced a remarkable synergistic effect on both cell lines, HCT-116 (IC_50_ = 0.1875 µM; CI = 0.43) and Caco-2 (IC_50_ = 0.08619 µM; CI = 0.25) cells [51].

Similarly, in the first-line treatment of late-stage HCC, where sorafenib is employed, its resistance steadily increases [140]. Ma et al. demonstrated that the combination of ACT and sorafenib enhanced the suppression of cell colony proliferation, migration of HCC cells, and angiogenesis of HUVECs. Furthermore, the synergistic effect of ACT and sorafenib was evidenced in vivo by their ability to inhibit the growth of JHH-7 xenografts [7]. Wen et al. found that ACT enhanced oxaliplatin (OXA), a primary chemotherapy drug for HCC, improving its effectiveness and reducing neurotoxicity by upregulating INPP4B and inhibiting the PI3K/AKT pathway [85].

In another study, ACT was effective against osteosarcoma (OS) [9], which is the most predominant pediatric bone cancer affecting children and young adults [141]. Cheimonidi et al. discovered that ACT exhibited toxicity across three OS cell lines (U2 OS (p53+/+, Rb1+/+), Sa OS (p53−/−, Rb1−/−), and KH OS) independent of their genetic background. In addition, they observed synergistic effects when 100 μM ACT was combined with 800 μM H_2_O_2_, 0.35 μM doxorubicin, and 10 nM epoxomicin (a proteasome inhibitor), effectively restoring sensitivity in doxorubicin-resistant OS cell lines [9].

### 3.2. Orientin in Synergy in Cancer

The combination of ORI and celecoxib (CLX), a COX-2 inhibitor with significant anti-inflammatory and antitumor effects, particularly in NSCLC, has shown considerable promise in lung cancer treatment [142]. Khalil et al. showed that ORI-CLX treatment had a superior anticancer effect, showing a higher apoptotic rate (44.2% vs. 22.1% with ORI alone). ORI-CLX effectively controlled the migration and invasion of A549 NSCLC cell lines by downregulating BCL-2, iNOS, and COX-2 expression. The metabolic regulator CYP-1A1 exhibited significant upregulation when treated with a combination of ORI (25 μM) and CLX (5 μM) compared to cells treated with ORI alone [91].

Another compound, curcumin (CUR), has diverse biological effects, including antioxidant, anti-inflammatory, and anticancer properties. Giordano and Tommonaro report that cancer is the primary focus in 37% of studies related to CUR [143]. Piwowarczyk and colleagues showed the synergistic impact of combining CUR and ORI within DOTAP: POPC liposomes. CUR + ORI of 10 µM (5 + 5 µM; 1.84 + 2.24 µg/mL) significantly induced caspase-3 in U-138 MG cells, and in T98G cells, a significant increase was seen with CUR + ORI at both 5 µM (2.5 + 2.5 µM; 0.92 + 1.12 µg/mL) and 10 µM (5 + 5 µM; 1.84 + 2.24 µg/mL). In liposomes, the combination of CUR (18.4 µg/mL) with ORI (22.4 µg/mL) showed synergistic cytotoxicity compared to ORI alone (44.8 µg/mL) with IC_50_ values of 12.5 ± 2.6 µg/mL for the combination and 12.0 ± 1.7 µg/mL for ORI in T98G cells after 24 h [70].

ORI enhances the sensitivity of CRC cells to 5FU by lowering its required dosage and alleviates 5FU-induced hepatotoxicity and nephrotoxicity. ORI effectively reduces 5FU-mediated HIF1α expression in connection with 5FU, resulting in its inhibition of CSC-driven tumor angiogenesis [144].

## 4. Acteoside and Orientin in Nanotechnology in Cancer

**Figure 4:**
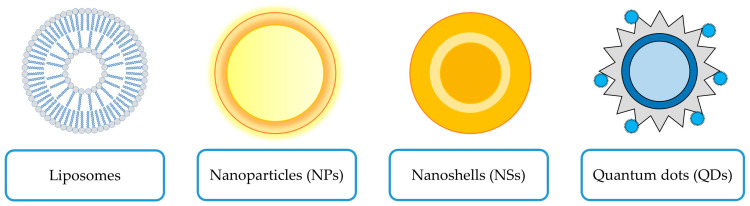
ACT and ORI in nanotechnology in the treatment of cancer.

Nanotechnology, gaining global attention for its promise in treating cancer, offers a fast, secure, economically feasible, and efficient approach [24]. Many anticancer drugs are often limited by their limited efficacy and potential side effects, including damage to healthy cells. Consequently, researchers are developing nanomaterials to improve drug efficacy and reduce toxicity [145]. This review focused on carriers containing metallic particles and liposomes belonging to LBDDSs and vesicular systems, because they have been used in ACT and ORI anticancer research. Figure 4 shows liposomes and metal-containing carriers such as NPs, NSs, and QDs used in previous studies for delivering ACT or ORI.

### 4.1. The Metal-Based Drug Delivery System

ACT and ORI can be delivered using metal-containing carriers such as NPs, NSs, and QDs. The first, metal-based NPs (M-NPs), comprise a diverse range of nanomaterials composited primarily consisting of essentially metal ions linked with other organic or inorganic components, inducing oxidative stress and leading to the death of cancer cells. The utilization of M-NPs in combination with drugs is promising in the treatment of breast, prostate, and brain cancers [146]. Commonly used metals in medical M-NPs include gold, silver, iron, nickel, cobalt, and some of their respective oxides [147]. Second carriers, metal NSs, feature a dielectric core, like silica, enveloped in an ultrathin metal shell, which is commonly gold. Gold NSs (GNSs) are used in biomedicine for cancer imaging and treatment [148,149]. Last, QDs, semiconductor nanocrystals with optoelectrical characteristics, emitting light energy of specific wavelengths upon photon excitation, exhibit value for bioimaging and targeted drug delivery in cancer treatment [150]. Core/shell QDs, a subtype, feature a core material encased in a shell of another substance [151]; for example, CdTe core/shell QDs, comprised of cadmium telluride (CdTe), a well-established nanomaterial known for its diverse properties, including anti-inflammatory effects [152].

### 4.2. The Lipid-Based Drug Delivery System

Among DDSs, LBDDSs excel in precise drug delivery for cancer treatment, addressing issues like systemic toxicity, drug resistance, and poor targeting associated with free drugs [153]. LBDDSs improve therapeutic outcomes, enhance bioavailability, protect drugs from degradation, enable controlled release, and reduce toxicity. They offer flexibility in lipid and additive composition and support various administration routes (oral, transdermal, parenteral, pulmonary) [153,154]. Additionally, LBDDSs can cross the blood–brain barrier (BBB) and reach target sites by their small size and ability to circumvent the reticular endothelial system [155].

This group represents liposomes as self-assembled spherical nanostructures consisting of a lipid bilayer, allowing for incorporating hydrophilic drugs in the aqueous core and hydrophobic drugs in the lipid bilayer [156,157]. For instance, Doxil (Caelyx), a PEG-coated liposomal form of doxorubicin with extended circulation and reduced toxicity, was the first liposome-based anticancer treatment approved by the US Food and Drug Administration (FDA) [158]. By 2019, sixteen liposome-based drugs were approved, including Myocet for breast cancer, Marqibo and Vyxeos for leukemia, Onivyde for pancreatic adenocarcinoma, and Mepact for osteosarcoma [159,160].

### 4.3. Acteoside in Nanotechnology in Cancer

Recent publications indicate a keen interest among researchers in integrating ACT into NPs, particularly examining the effects of gold and nickel on the proliferation of the doxorubicin-resistant K562 human immortalized myelogenous leukemia cell line [161,162]. Zhang et al. used gold NPs (Au-NPs) to demonstrate the suppressive impact of ACT-loaded Au-NPs on K562 cells in vitro and in vivo. They found that ACT-loaded Au-NPs significantly reduced K562 cell viability and induced tumor cell apoptosis compared to the same concentrations of Au-NPs or ACT alone. Moreover, in a murine model featuring implanted tumors, the intravenous administration of ACT-loaded gold NPs inhibited tumor growth and prompted apoptosis in cancer cells [161]. On the other hand, Chen et al. developed multifunctional ACT-coated nickel NPs (ACT-Ni), which, compared to ACT alone, induced apoptosis and inhibited the proliferation of K562 cells in vitro and in vivo. Additionally, an in vivo investigation demonstrated that the administration of ACT-Ni effectively suppressed the growth of tumors in mice [162]. Interestingly, Chittasupho et al. compared the cytotoxicity of C. *chinense* stem extract (abundant in ACT and isoverbascoside) with its nanoparticle-encapsulated form across cancer cell lines. The IC_50_ values for stem extract alone were 109.2 µg/mL for MCF-7, 155.6 µg/mL for HeLa, 206.9 µg/mL for A549, and 423 µg/mL for SKOV-3 cells, while the nanoparticle-encapsulated extract showed higher IC_50_ values of 1664 µg/mL for MCF-7, 1363 µg/mL for HeLa, 2044 µg/mL for A549, and 1905 µg/mL for SKOV-3 cancer cell lines. MCF-7 cells were most sensitive to the extract, while the NPs maintained their anticancer mechanism despite reduced cytotoxicity. Extract and NPs reduced colony formation, induced apoptosis and necrosis, reduced mitochondrial membrane potential, and triggered G_0_/G_1_ phase arrest in MCF-7 cells [163].

An instance showcasing the utilization of gold NSs (GNSs) to deliver ACT is novel DDSs, comprising poly(N-isopropyl acrylamide) (PNIPAM) microsphere cores coated with GNSs, together with the ACT, which induced apoptosis in drug-resistant leukemia cells (K562/A02; KA) within xenografted tumors in KA nude mice [164]. Other researchers utilized QDs to deliver ACT. Zhao et al. used highly luminescent and stable CdTe core/shell cadmium telluride QDs for the long-term intracellular delivery of ACT. The apoptosis rate in adriamycin-resistant human hepatoma HepG2 cells (HepG2/ADM cells) rose when administered ACT-QDs compared to treatment with CdTe QDs or ACT alone [165].

However, from the CUR, BDMC, ORI, and ACT encapsulated in a liposomal nanoformula of two lipid types (DOTAP: POPC), ACT in liposomes exhibited the highest activity against the T98G GBM cell lines. The liposomal encapsulation of ACT (0.25–5 μM) resulted in a dose-dependent increase in the protein levels of p53 and caspase-3, which are fundamental effectors associated with apoptosis. Furthermore, compounds’ positively charged liposomal encapsulation facilitates passage across the BBB. Interestingly, ACT alone showed IC_50_ values of 85.0 ± 4.3 µM in T98G and 44.0 ± 4.1 µM in U-138 MG cells, while liposomal encapsulation greatly enhanced potency, lowering IC_50_ to 2.9 ± 0.9 µM and 4.0 ± 1.1 µM, respectively. Additionally, the encapsulation of ACT combined with BDMC in liposomes (ACT:BDMC:POPC:DOTAP ratio of 0.05:0.05:8:2, 15.4 + 31.2 µg/mL BDMC + ACT) reduced the IC_50_ from >100 µM to 23.0 ± 1.8 µM in T98G cells and from 69.0 ± 5.2 µM to 11.0 ± 1.4 µM in U-138 MG cells. [70]. This highlights the enhanced efficacy of the liposomal formulation in delivering these compounds to the GBM cells compared to their direct combination.

### 4.4. Orientin in Nanotechnology in Cancer

Another study employed NPs for the delivery of ORI. Srećković et al. synthesized silver NPs (AgNPs) using aqueous extracts of *Lythrum salicaria* L., both aerial parts (LSA-AgNPs) and root extract (LSR-AgNPs), which were rich in ORI, vitexin, and isovitexin. LSA-AgNPs exhibited IC_50_ values of 20.5 ± 5 and 12.7 ± 6 μg/mL against A431 (epidermoid carcinoma) and SVT2 (transformed fibroblast) cells, respectively. In contrast, LSR-AgNPs showed efficacy solely against A431 cancer cells with IC_50_ values of 62 ± 17 μg/mL [166].

Liposomal delivery enhances the efficacy of ORI efficacy in T98G and U-138 MG cells, reducing IC_50_ values from >100 µM to 12.0 ± 1.7 µM (T98G) and 7.0 ± 1.5 µM (U-138 MG). Liposomal nanoformulation (CUR:ORI:POPC:DOTAP (0.05:0.05:8:2) 18.4 + 22.4 µg/mL CUR + ORI significantly enhanced the synergistic cytotoxicity of CUR and ORI, reducing IC_50_ from 24.0 ± 2.3 µM to 13.0 ± 1.4 µM in U-138 MG cells and from >100 µM to 12.5 ± 2.6 µM in T98G cells [70]. 

Table 3 summarizes (a) the sources, mechanisms, target cancers, and synergies of ACT and ORI and (b) the available research on delivering ACT and ORI into DDSs in the context of cancer treatment.

## 5. Barriers to Commercialization

Despite robust in vitro and in vivo evidence of anticancer activity, there are currently no active, registered clinical trials investigating ACT and ORI in oncological therapy. There is a critical need for further studies on their bioavailability, including poor absorption, low bioaccessibility, and efflux transport, as well as pharmacokinetics and toxicity to establish a complete drug profile suitable for clinical trials and eventual commercialization [70,167,168,169]. Key barriers to commercialization include challenges in standardizing these compounds, ensuring pharmaceutical-grade quality and adequate supply, and securing financial investment and interest from the pharmaceutical industry. Liu et al. demonstrated the feasibility of scaling up ORI production using a coupled catalytic strategy [170]. For ACT, scale-up production is possible through bioreactor systems, which promote the growth of *R. glutinosa* adventitious roots, increasing ACT content and antioxidant activity [171]. Moreover, glycosides such as ACT and ORI exhibit significant variability in purity and stability, emphasizing the need for advanced DDSs to improve their production and delivery [172,173,174], although this approach may increase production costs.

## 6. Conclusions

ACT and ORI have anticancer properties, which are confirmed by numerous studies. Notably, the scope of research on ACT in cancer currently exceeds that on ORI, which highlights that the anticancer effects of ORI are so far less known. Available studies demonstrate the impact of both substances on liver cancer with evidence suggesting a synergistic effect between ACT and sorafenib, which is a drug commonly used in the first-line treatment of advanced HCC.

One of the significant challenges in using these compounds is their low bioavailability, which hinders their therapeutic potential. Advanced methods, such as nanotechnology and innovative DDSs, offer potential solutions to this issue, paving the way for using these substances in anticancer therapy. These methods aid in improving the bioavailability of compounds and facilitate targeted therapies, thereby minimizing adverse effects on healthy body cells. Integrating these natural compounds with advanced technologies holds promise for more effective cancer treatments, marking a promising medical prospect. Furthermore, it included publications with synergy, suggesting that the combined effect of these compounds with others could bring substantial benefits for achieving even more effective anticancer effects and offer a promising avenue for overcoming drug resistance. These innovative approaches set the stage for future research on natural compounds, such as ACT and ORI, utilizing nanotechnology and exploring the synergistic interaction of natural compounds against cancer.

## Figures and Tables

**Table 1:** The IC_50_ values of ACT in various types of cancer.

ACT				
Types of Cancer	Cell Line	IC_50_ [µM]	Incubation Time [h]	References
Breast cancer	MCF-7	85.30 ± 12.6	48	[38]
	MCF-7	0.624 ± 0.024	48	[40]
	MCF-7	219.1 ± 1.40	24	[41]
		154.2 ± 2.71	48	
		113.1 ± 2.81	72	
	MCF-7	0.127	24	[42]
		0.2174	48	
		0.2828	72	
	MCF-7	215.86	24	[39]
	MDA-MB-231	312.2 ± 3.28	24	[41]
		244.9 ± 4.96	48	
		200.2 ± 2.45	72	
	MDA-MB-231	0.1597	24	[42]
		0.2584	48	
		0.2563	72	
	4 T1	116.7	24	[43]
Colorectal cancer, colon cancer	HT-29	120.40 ± 6.58	48	[38]
	HT-29	1.489 ± 0.096	48	[40]
	HT-29	144.5	24	[53]
		108.82	48	
		66.68	72	
	Caco-2	507.6 ± 4.05	24	[41]
		466.9 ± 8.71	48	
		280.3 ± 6.13	72	
	SNU-C5	73.6 ± 4.9	24	[56]
	HCT-116	208.89	24	[53]
		97.86	48	
		63.51	72	
	HCT-116	>100	24	[54]
	LoVo	83.83	24	[53]
		59.62	48	
		43.96	72	
	SW620	52.73	24	[53]
		42.42	48	
		29.05	72	
Brain cancer	U-138 MG	274.3 ± 2.61	24	[41]
		201.9 ± 4.90	48	
		156.6 ± 4.74	72	
	U-138 MG	44.0 ± 4.1	24	[70]
	U251-MG	3152.7 ± 4.15	24	[41]
		2412.5 ± 7.97	48	
		1165.3 ± 6.05	72	
	T98G	85.0 ± 4.3	24	[70]
Liver cancer	HepG2	261.3 ± 3.52	24	[41]
		219.6 ± 2.65	48	
		173.8 ± 1.59	72	
	HepG2	>100	24	[54]
	HepG2	53.1 ± 6.9	24	[56]
	dRLh-84	99.23	48	[76]
Lung cancer	A549	114.57 ± 8.53	48	[38]
	A549	93.92 ± 2.72	24	[54]
	A549	397.70 ± 25.33	24	[90]
	3LL Lewis	62.5 ± 4.6	24	[56]
Leukemia	HL-60	26.7	48	[105]
	HL-60	38.3 ± 1.3	24	[56]
Cervical cancer	HeLa	>320.20	48	[76]
Gastric cancer	MGc80-3	45.92 ± 4.48	24	[54]
Ovarian cancer	OVCAR-3	314.1 ± 3.52	24	[41]
		232.0 ± 5.08	48	
		162.8 ± 3.41	72	
Sarcoma	S-180	47.39	48	[76]
Lymphoid tumor cell line	P-388/D1	353.83	48	[76]
	U-937	42.5 ± 2.1	24	[56]

**Table 2:** The IC_50_ values of ORI in various types of cancer.

ORI				
Types of Cancer	Cell Line	IC_50_ [µM]	Incubation Time [h]	References
Breast cancer	MCF-7	>178.42	24	[44]
Colorectal cancer, colon cancer	CaCo-2	>178.42	24	[44]
	CaCo-2	190.86	48	[57]
Brain cancer	U87 MG	68.94	48	[57]
Cervical cancer	HeLa	>178.42	24	[44]

**Table 3:** (a) The sources, mechanisms, target cancers, and synergies for ACT and ORI, and (b) available research about delivering ACT and ORI into DDSs in the context of cancer treatment.

(a) Main Information	ACT	ORI
Sources	*Oleaceae*, *Bignoniaceae*, *Verbenaceae, Labiatae* [6,7]	*Ocimum sanctum*, *Commelina communis*, *Jatropha gossypifolia, Phyllostachys nigra* [10]
Key mechanisms	Increasing Bax expression; decreasing Bcl-2 expression [43] Changes Bax/Bcl-2 ratio [52]. Inhibiting MMP-9 transcription (via NF-κB/AP-1) [34] Modulating the PD-1 checkpoint and PD-L1 expression [39] Increasing caspase-3 [43] Increasing HIPK2, p53 [53] Increasing ROS levels [38] Suppressing Rac-1, HIF-1α, Zeb-1 signaling pathway [55] Reducing c-Met, EMT markers (snail, vimentin, zeb1) [66] Activates SHP-1, suppressing STAT3 phosphorylation [67] Suppressing HMGA2, inhibiting Wnt/β-catenin signaling [8] Inhibiting CDC42 activation via HMGB1/RAGE signaling [119] Inhibiting NFκB/IκB signaling pathway [75] Increasing p53, reducing KLK1, KLK2, KLK4, KLK9, KLK10 gene levels [7] Reducing CCL20, enhancing apoptosis via ERK1/2 pathway [77] Inducing G1 arrest, upregulating p21^CIP1/WAF1^, p27^KIP1^ levels and decreasing CDK2, CDK4 and CDK6 activities [56] Inducing M1 macrophages polarization [125] Blocking METTL3-regulated miR-31-5p/HIPK2 axis [127]	Increasing Bax expression; decreasing Bcl-2 expression; increasing Bax/Bcl-2 ratio, activating caspase-3, caspase-9 [114] Suppressing the expression of MMP-9 and IL-8 [46] Inducing G_0_/G_1_ cell cycle arrest [59] Decreasing NF-κB, TNF-α, IL-6 expression, downregulating iNOS, COX-2 overexpression [61] Increasing p53 [120]
Target cancers	Breast cancer [34,38] Colorectal cancer, colon cancer [52,53] Brain cancer [66,67] Liver cancer [7,75] Lung cancer [38,90] Skin cancer [9,96] Leukemia [56,106] Cervical cancer [76,98] Esophageal cancer [119] Prostate cancer [121] Gastric cancer [124] Ovarian cancer [41] Oral cancer [126] Sarcoma [128] Lymphoid tumor cells [76]	Breast cancer [44,45] Colorectal cancer, colon cancer [44,57] Brain cancer [57] Liver cancer [78,79] Lung cancer [91] Skin cancer [100] Leukemia [107] Cervical cancer [44,114] Esophageal cancer [120] Bladder carcinoma [129]
Optimal synergies	+ TMZ (Brain cancer) [137] + BDMC (Brain cancer) [70] + 5-FU (Colorectal cancer, colon cancer) [139] + Sorafenib (Liver cancer) [7] +H_2_O_2_, epoxomicin, doxorubicin (Osteosarcoma) [9] + OXA (Hepatocellular carcinoma) [85]	+ CLX (Lung cancer) [91] + CUR (Brain cancer) [70] + 5-FU (Colorectal cancer) [144]
**(b) DDSs**	**ACT**	**ORI**
Nanoparticles (NPs)	ACT-Au: 2 mg ACT and 30 mg gold NPs (Au). ACT-Au reduced K562 cell viability, ACT-Au promoted apoptosis (about 45%) more than Au (less than 20%) or ACT (less than 30%). ACT-Au injection in mice suppressed tumor growth, induced apoptosis [161]. ACT-Ni: 2mg ACT and 30 mg Ni nanoparticles. ACT-Ni treatment significantly reduced tumor volume and weight with more pronounced apoptosis than the ACT. ACT and Ni, bound by electrostatic interaction, effectively inhibited tumor growth. ACT-Ni treatment suppressed tumor growth in mice [162]. *C. chinense* stem extract, containing ACT and isoverbascoside, in NPs. At 500 µg/mL, the extract reduced MCF-7 colony formation to 1.40 ± 1.09% and induced 12.13% late-apoptotic cells, while NPs at the same concentration completely inhibited colony formation and induced 25.57% apoptotic cells in MCF-7 cells. The extract and NPs contained 553.20 ± 68.74 and 490.26 ± 24.12 mg GAE per gram, respectively. NPs enhance compounds’ solubility and stability, improving bioavailability [163].	The aqueous extracts of *L. salicaria* aerial part (LSA) and root (LSR), used for AgNP synthesis, contained total phenolic compounds (among others ORI) at concentrations of 99.56 and 26.44 mg GAE per g of dry plant weight, respectively. LSA-AgNPs had IC_50_ values of 20.5 ± 5 μg/mL against A431 and 12.7 ± 6 μg/mL for SVT2 cells. LSR-AgNPs were only effective against A431 cells with an IC_50_ of 62 ± 17 μg/mL. The alkaline environment boosts the negative charge on phenolic groups, aiding the faster reduction and stabilization of AgNPs. ORI in extracts likely contributes to AgNP formation and stability [166].
Nanoshells (NSs)	ACT-PMS: ACT + PNIPAM/gold NSs (GNSs) structures (PMS): 2 mg VB and 30 mg PMS. Compared to PMS or ACT alone, PMS enhanced apoptosis and inhibited tumor growth in KA cells. ACT-PMS activated the expression of apoptosis-related caspase proteins. PMS structures enable efficient drug release due to their swelling and shrinking capabilities. ACT’s hydrophobicity was addressed by ACT-PMS, offering a reference for ACT’s application. ACT-PMS treatment increased apoptosis in KA cells, boosting ACT’s efficiency [164].	no data
Quantum dots (QDs)	In HepG2/ADM cells, the apoptotic cell percentages were 65.6% for ACT–QDs, 34.3% for ACT, and 11.6% for the control (no treatment). ACT content in ACT–QDs was 18.3 wt.%, equivalent to 183 mg of ACT per gram of ACT–QDs. ACT–QDs were stable after one year of storage in a sealed bottle at ambient conditions. ACT–QDs caused nanoscale holes in living cells, potentially improving drug delivery across the cell membrane [165].	no data
Liposomes	ACT:POPC: DOTAP (0.1:8:2) containing 62.5 µg/mL ACT. Liposomal delivery of ACT reduced IC_50_ values from 85.0 ± 4.3 µM to 2.9 ± 0.9 µM (T98G) and from 44.0 ± 4.1 µM to 4.0 ± 1.1 µM (U-138 MG). Encapsulation efficiency of ACT was 99.2 ± 1.8% [70].	ORI:POPC: DOTAP (0.1:8:2) containing 44.8 µg/mL ORI. Liposomal delivery of ORI reduced IC_50_ values from >100 µM to 12.0 ± 1.7 µM (T98G) and 7.0 ± 1.5 µM (U-138 MG). Encapsulation efficiency of ORI was 79.3 ± 1.4% [70].

## Abbreviations

The following abbreviations are used in this manuscript:

5-FU	5-Fluorouracil
ACF	Aberrant crypt foci
ACT	Acteoside
ARE	Antioxidant response element
BC	Breast cancer
BCSCs	Breast cancer stem cells
BBB	Blood–brain barrier
BDMC	Bisdemethoxycurcumin
CC	Colon cancer
CDK	Cyclin-dependent kinase
CdTe	Cadmium telluride
CLX	Celecoxib
CNS	Central nervous system
CRC	Colorectal cancer
CUR	Curcumin
DDSs	Drug delivery systems
DHT	Dihydrotestosterone
DMH	1,2-Dimethylhydrazine
EC	Esophageal cancer
EAC	Esophageal adenocarcinoma
ED_50_	Effective dose
EGFR	Epidermal growth factor receptor
EMT	Epithelial-to-mesenchymal transition
ERβ	Estrogen receptor beta
ERK	Extracellular signal-regulated kinase
ESCC	Esophageal squamous cell carcinoma
GBM	Glioblastoma multiforme
GNSs	Gold nanoshells
HCC	Hepatocellular carcinoma
HMGA2	High mobility group A2
HMGB1	High mobility group box 1
HPV	Human papillomavirus
IL-8	Interleukin-8
LBDDSs	Lipid-based drug delivery systems
MITF	Microphthalmia-associated transcription factor
MMP-9	Matrix metalloproteinase-9
MyD88	Myeloid differentiation primary response 88N
Ps	Nanoparticles
Nrf2	Nuclear factor erythroid 2-related factor 2)
NSCLC	Non-small cell lung cancer
NSs	Nanoshells
OC	Ovarian cancer
ORI	Orientin
OS	Osteosarcoma
OSCC	Oral squamous cell carcinoma
OXA	Oxaliplatin
P-gp	P-glycoprotein
PI3K	Phosphatidylinositol 3-kinase
PMA	Phorbol-12-myristate-13-acetate
p-STAT3	Phosphorylated signal transducer and activator of transcription 3
PSA	Prostate-specific antigen
QDs	Quantum dots
RAGE	Receptor for advanced glycosylation end-products
ROS	Reactive oxygen species
SarA	Staphylococcal accessory regulator
SCC	Squamous cell carcinoma
SCLC	Small cell lung cancer
SFE-CO_2_	Supercritical fluid extraction
SHP-1	Protein tyrosine phosphatase 1
SI	Selectivity index
TLR4	Toll-like receptor 4
TMZ	Temozolomide
TPA	12-O-Tetradecanoylphorbol-13-acetate
TRP-1	Tyrosinase-related protein-1

## References

[1] Chen T., Ren L., Liu X., Zhou M., Li L., Xu J., Zhu X. (2018). DNA Nanotechnology for Cancer Diagnosis and Therapy. Int. J. Mol. Sci..

[2] Siegel R.L., Giaquinto A.N., Jemal A. (2024). Cancer Statistics, 2024. CA Cancer J. Clin..

[3] Miura K., Satoh M., Kinouchi M., Yamamoto K., Hasegawa Y., Kakugawa Y., Kawai M., Uchimi K., Aizawa H., Ohnuma S. (2015). The Use of Natural Products in Colorectal Cancer Drug Discovery. Expert Opin. Drug Discov..

[4] Basmadjian C., Zhao Q., Bentouhami E., Djehal A., Nebigil C.G., Johnson R.A., Serova M., de Gramont A., Faivre S., Raymond E. (2014). Cancer Wars: Natural Products Strike Back. Front. Chem..

[5] Muller A.G., Sarker S.D., Saleem I.Y., Hutcheon G.A. (2019). Delivery of Natural Phenolic Compounds for the Potential Treatment of Lung Cancer. DARU J. Pharm. Sci..

[6] Alipieva K., Korkina L., Orhan I.E., Georgiev M.I. (2014). Verbascoside—A Review of Its Occurrence, (Bio)Synthesis and Pharmacological Significance. Biotechnol. Adv..

[7] Ma D., Wang J., Liu L., Chen M., Wang Z. (2020). Acteoside as a Potential Therapeutic Option for Primary Hepatocellular Carcinoma: A Preclinical Study. BMC Cancer.

[8] Jia W.-Q., Zhu J.-W., Yang C.-Y., Ma J., Pu T.-Y., Han G.-Q., Zou M.-M., Xu R.-X. (2020). Verbascoside Inhibits Progression of Glioblastoma Cells by Promoting Let-7g-5p and down-Regulating HMGA2 via Wnt/Beta-Catenin Signalling Blockade. J. Cell. Mol. Med..

[9] Cheimonidi C., Samara P., Polychronopoulos P., Tsakiri E.N., Nikou T., Myrianthopoulos V., Sakellaropoulos T., Zoumpourlis V., Mikros E., Papassideri I. (2018). Selective Cytotoxicity of the Herbal Substance Acteoside against Tumor Cells and Its Mechanistic Insights. Redox Biol..

[10] Lam K.Y., Ling A.P.K., Koh R.Y., Wong Y.P., Say Y.H. (2016). A Review on Medicinal Properties of Orientin. Adv. Pharmacol. Sci..

[11] Thangaraj K., Natesan K., Palani M., Vaiyapuri M. (2018). Orientin, a Flavanoid, Mitigates 1, 2 Dimethylhydrazine-Induced Colorectal Lesions in Wistar Rats Fed a High-Fat Diet. Toxicol. Rep..

[12] Yücer R., Schröder A., Topçu G., Efferth T. (2025). Identification of Anti-Inflammatory and Anti-Cancer Compounds Targeting the NF-κB-NLRP3 Inflammasome Pathway from a Phytochemical Library of the Sideritis Genus. J. Ethnopharmacol..

[13] Vaziri-Amjad S., Rahgosha R., Taherkhani A. (2024). Potential JAK2 Inhibitors from Selected Natural Compounds: A Promising Approach for Complementary Therapy in Cancer Patients. Evid.-Based Complement. Altern. Med. ECAM.

[14] Yang J., Hua Z., Zheng Z., Ma X., Zhu L., Li Y. (2023). Acteoside Inhibits High Glucose-Induced Oxidative Stress Injury in RPE Cells and the Outer Retina through the Keap1/Nrf2/ARE Pathway. Exp. Eye Res..

[15] Li M., Zhou F., Xu T., Song H., Lu B. (2018). Acteoside Protects against 6-OHDA-Induced Dopaminergic Neuron Damage via Nrf2-ARE Signaling Pathway. Food Chem. Toxicol. Int. J. Publ. Br. Ind. Biol. Res. Assoc..

[16] Vasudevan Sajini D., Thaggikuppe Krishnamurthy P., Chakkittukandiyil A., Mudavath R.N. (2024). Correction: Orientin Modulates Nrf2-ARE, PI3K/Akt, JNK-ERK1/2, and TLR4/NF-κB Pathways to Produce Neuroprotective Benefits in Parkinson’s Disease. Neurochem. Res..

[17] Di Lorenzo C., Colombo F., Biella S., Stockley C., Restani P. (2021). Polyphenols and Human Health: The Role of Bioavailability. Nutrients.

[18] Zhang W., Huo S.-X., Wen Y.-L., Xing H., Zhang Q., Li N., Zhao D., Sun X.-L., Xu J., Yan M. (2015). Pharmacokinetics of Acteoside Following Single Dose Intragastric and Intravenous Administrations in Dogs. Chin. J. Nat. Med..

[19] Wu Y.-T., Lin L.-C., Sung J.-S., Tsai T.-H. (2006). Determination of Acteoside in *Cistanche Deserticola* and *Boschniakia Rossica* and Its Pharmacokinetics in Freely-Moving Rats Using LC–MS/MS. J. Chromatogr. B.

[20] Zhou F., Huang W., Xu T., Wu L., Chen Q., Peng J., Liu X., Lu B. (2020). Natural P-Gp Inhibitor EGCG Improves the Acteoside Absorption in Caco-2 Cell Monolayers and Increases the Oral Bioavailability of Acteoside in Rats. Food Chem. Toxicol..

[21] Li D., Wang Q., Yuan Z.F., Zhang L., Xu L., Cui Y., Duan K. (2008). Pharmacokinetics and Tissue Distribution Study of Orientin in Rat by Liquid Chromatography. J. Pharm. Biomed. Anal..

[22] Zhang Y., Tie X., Bao B., Wu X., Zhang Y. (2007). Metabolism of Flavone C-Glucosides and p-Coumaric Acid from Antioxidant of Bamboo Leaves (AOB) in Rats. Br. J. Nutr..

[23] Wang S., Su R., Nie S., Sun M., Zhang J., Wu D., Moustaid-Moussa N. (2014). Application of Nanotechnology in Improving Bioavailability and Bioactivity of Diet-Derived Phytochemicals. J. Nutr. Biochem..

[24] Dessale M., Mengistu G., Mengist H.M. (2022). Nanotechnology: A Promising Approach for Cancer Diagnosis, Therapeutics and Theragnosis. Int. J. Nanomed..

[25] Wang Q., Atluri K., Tiwari A.K., Babu R.J. (2023). Exploring the Application of Micellar Drug Delivery Systems in Cancer Nanomedicine. Pharmaceuticals.

[26] Cheng C.-T., Castro G., Liu C.-H., Lau P. (2019). Advanced Nanotechnology: An Arsenal to Enhance Immunotherapy in Fighting Cancer. Clin. Chim. Acta Int. J. Clin. Chem..

[27] Zhu Y., Wang M., Zhang J., Peng W., Firempong C.K., Deng W., Wang Q., Wang S., Shi F., Yu J. (2015). Improved Oral Bioavailability of Capsaicin via Liposomal Nanoformulation: Preparation, in Vitro Drug Release and Pharmacokinetics in Rats. Arch. Pharmacal Res..

[28] Li C., Zhang Y., Su T., Feng L., Long Y., Chen Z. (2012). Silica-Coated Flexible Liposomes as a Nanohybrid Delivery System for Enhanced Oral Bioavailability of Curcumin. Int. J. Nanomed..

[29] Shaikh J., Ankola D.D., Beniwal V., Singh D., Kumar M.N.V.R. (2009). Nanoparticle Encapsulation Improves Oral Bioavailability of Curcumin by at Least 9-Fold When Compared to Curcumin Administered with Piperine as Absorption Enhancer. Eur. J. Pharm. Sci. Off. J. Eur. Fed. Pharm. Sci..

[30] Shrestha H., Bala R., Arora S. (2014). Lipid-Based Drug Delivery Systems. J. Pharm..

[31] Pagar K.R., Khandbahale S.V. (2019). A Review on Novel Drug Delivery System: A Recent Trend. Asian J. Pharm. Technol..

[32] Bray F., Laversanne M., Weiderpass E., Soerjomataram I. (2021). The Ever-Increasing Importance of Cancer as a Leading Cause of Premature Death Worldwide. Cancer.

[33] Antonova L., Aronson K., Mueller C.R. (2011). Stress and Breast Cancer: From Epidemiology to Molecular Biology. Breast Cancer Res. BCR.

[34] Liao Y.-F., Rao Y.K., Tzeng Y.-M. (2012). Aqueous Extract of Anisomeles Indica and Its Purified Compound Exerts Anti-Metastatic Activity through Inhibition of NF-κB/AP-1-Dependent MMP-9 Activation in Human Breast Cancer MCF-7 Cells. Food Chem. Toxicol..

[35] Barzaman K., Karami J., Zarei Z., Hosseinzadeh A., Kazemi M.H., Moradi-Kalbolandi S., Safari E., Farahmand L. (2020). Breast Cancer: Biology, Biomarkers, and Treatments. Int. Immunopharmacol..

[36] Bilynskyj B.T. (2010). The Breast Cancer Treatment as a Marker of Progress in Oncology. Exp. Oncol..

[37] Comşa Ş., Cîmpean A.M., Raica M. (2015). The Story of MCF-7 Breast Cancer Cell Line: 40 Years of Experience in Research. Anticancer Res..

[38] Vasincu A., Neophytou C.M., Luca S.V., Skalicka-Woźniak K., Miron A., Constantinou A.I. (2020). 6-O-(3″,4″-Di-O-Trans-Cinnamoyl)-α-l-Rhamnopyranosylcatalpol and Verbascoside: Cytotoxicity, Cell Cycle Kinetics, Apoptosis, and ROS Production Evaluation in Tumor Cells. J. Biochem. Mol. Toxicol..

[39] Cabatit K.A., Carandang L.J., Saragpon D.J., Minalang K., Paulin J., Devanadera M.K., Daya M. (2025). Assessment of Cytotoxicity, Impact on Cell Migration and Apoptotic Modulation of Acteoside and Plantamajoside on Human Breast Adenocarcinoma (MCF-7). Asian Pac. J. Cancer Prev..

[40] Delazar A., Asnaashari S., Nikkhah E., Asgharian P. (2019). Phytochemical Analysis and Antiproliferative Activity of the Aerial Parts of Scrophularia Subaphylla. Res. Pharm. Sci..

[41] Budzianowska A., Totoń E., Romaniuk-Drapała A., Kikowska M., Budzianowski J. (2023). Cytotoxic Effect of Phenylethanoid Glycosides Isolated from *Plantago lanceolata* L.. Life.

[42] Şenol H., Tulay P., Ergören M.Ç., Hanoğlu A., Çalış İ., Mocan G. (2021). Cytotoxic Effects of Verbascoside on MCF-7 and MDA-MB-231. Turk. J. Pharm. Sci..

[43] Daneshforouz A., Nazemi S., Gholami O., Kafami M., Amin B. (2021). The Cytotoxicity and Apoptotic Effects of Verbascoside on Breast Cancer 4T1 Cell Line. BMC Pharmacol. Toxicol..

[44] Schuster R., Holzer W., Doerfler H., Weckwerth W., Viernstein H., Okonogi S., Mueller M. (2016). Cajanus Cajan—A Source of PPARγ Activators Leading to Anti-Inflammatory and Cytotoxic Effects. Food Funct..

[45] Mohammed R.S., Abou Zeid A.H., El Hawary S.S., Sleem A.A., Ashour W.E. (2014). Flavonoid Constituents, Cytotoxic and Antioxidant Activities of *Gleditsia triacanthos* L. Leaves. Saudi J. Biol. Sci..

[46] Kim S.-J., Pham T.-H., Bak Y., Ryu H.-W., Oh S.-R., Yoon D.-Y. (2018). Orientin Inhibits Invasion by Suppressing MMP-9 and IL-8 Expression via the PKCα/ ERK/AP-1/STAT3-Mediated Signaling Pathways in TPA-Treated MCF-7 Breast Cancer Cells. Phytomedicine Int. J. Phytother. Phytopharm..

[47] Zu Y., Liu X., Fu Y., Wu N., Kong Y., Wink M. (2010). Chemical Composition of the SFE-CO2 Extracts from Cajanus Cajan (L.) Huth and Their Antimicrobial Activity in Vitro and in Vivo. Phytomedicine.

[48] Czemplik M., Mierziak J., Szopa J., Kulma A. (2016). Flavonoid C-Glucosides Derived from Flax Straw Extracts Reduce Human Breast Cancer Cell Growth In Vitro and Induce Apoptosis. Front. Pharmacol..

[49] Paschke S., Jafarov S., Staib L., Kreuser E.-D., Maulbecker-Armstrong C., Roitman M., Holm T., Harris C.C., Link K.-H., Kornmann M. (2018). Are Colon and Rectal Cancer Two Different Tumor Entities? A Proposal to Abandon the Term Colorectal Cancer. Int. J. Mol. Sci..

[50] Katsaounou K., Nicolaou E., Vogazianos P., Brown C., Stavrou M., Teloni S., Hatzis P., Agapiou A., Fragkou E., Tsiaoussis G. (2022). Colon Cancer: From Epidemiology to Prevention. Metabolites.

[51] Attia Y.M., El-Kersh D.M., Wagdy H.A., Elmazar M.M. (2018). Verbascoside: Identification, Quantification, and Potential Sensitization of Colorectal Cancer Cells to 5-FU by Targeting PI3K/AKT Pathway. Sci. Rep..

[52] Firat F., Türkoğlu C., Ozdal Kurt F., Vatansever H.S. (2022). Is Acteoside Effects on Colon Cancer Stem Cells Via Inflamation or Apoptosis?. Genel Tıp Derg..

[53] Zhou L., Feng Y., Jin Y., Liu X., Sui H., Chai N., Chen X., Liu N., Ji Q., Wang Y. (2014). Verbascoside Promotes Apoptosis by Regulating HIPK2–P53 Signaling in Human Colorectal Cancer. BMC Cancer.

[54] Xiang Y., Jing Z., Haixia W., Ruitao Y., Huaixiu W., Zenggen L., Lijuan M., Yiping W., Yanduo T. (2017). Antiproliferative Activity of Phenylpropanoids Isolated from Lagotis Brevituba Maxim. Phytother. Res..

[55] Nabiuni M., Seyfi D., Behzad S., Parivar K., Tahmaseb M., Amini E. (2018). Verbascoside Attenuates Rac-1 and HIF-1α Signaling Cascade in Colorectal Cancer Cells. Anticancer Agents Med. Chem..

[56] Lee K.-W., Kim H.J., Lee Y.S., Park H.-J., Choi J.-W., Ha J., Lee K.-T. (2007). Acteoside Inhibits Human Promyelocytic HL-60 Leukemia Cell Proliferation via Inducing Cell Cycle Arrest at G 0 /G 1 Phase and Differentiation into Monocyte. Carcinogenesis.

[57] Pirvu L.C., Pintilie L., Albulescu A., Stefaniu A., Neagu G. (2023). Anti-Proliferative Potential of Cynaroside and Orientin—In Silico (DYRK2) and In Vitro (U87 and Caco-2) Studies. Int. J. Mol. Sci..

[58] Fayed M.A.A., Abouelela M.E., Refaey M.S. (2022). Heliotropium Ramosissimum Metabolic Profiling, in Silico and in Vitro Evaluation with Potent Selective Cytotoxicity against Colorectal Carcinoma. Sci. Rep..

[59] Thangaraj K., Balasubramanian B., Park S., Natesan K., Liu W., Manju V. (2019). Orientin Induces G0/G1 Cell Cycle Arrest and Mitochondria Mediated Intrinsic Apoptosis in Human Colorectal Carcinoma HT29 Cells. Biomolecules.

[60] Thangaraj K., Natesan K., Settu K., Palani M., Govindarasu M., Subborayan V., Vaiyapuri M. (2018). Orientin Mitigates 1, 2-Dimethylhydrazine Induced Lipid Peroxidation, Antioxidant and Biotransforming Bacterial Enzyme Alterations in Experimental Rats. J. Cancer Res. Ther..

[61] Thangaraj K., Vaiyapuri M. (2017). Orientin, a C-Glycosyl Dietary Flavone, Suppresses Colonic Cell Proliferation and Mitigates NF-κB Mediated Inflammatory Response in 1,2-Dimethylhydrazine Induced Colorectal Carcinogenesis. Biomed. Pharmacother. Biomedecine Pharmacother..

[62] Tang M., Rich J.N., Chen S. (2021). Biomaterials and 3D Bioprinting Strategies to Model Glioblastoma and the Blood–Brain Barrier. Adv. Mater..

[63] McLendon R.E., Halperin E.C. (2003). Is the Long-Term Survival of Patients with Intracranial Glioblastoma Multiforme Overstated?. Cancer.

[64] Iturrioz-Rodríguez N., Bertorelli R., Ciofani G. (2021). Lipid-Based Nanocarriers for The Treatment of Glioblastoma. Adv. Nanobiomed Res..

[65] Hottinger A.F., Stupp R., Homicsko K. (2014). Standards of Care and Novel Approaches in the Management of Glioblastoma Multiforme. Chin. J. Cancer.

[66] Hei B., Wang J., Wu G., Ouyang J., Liu R. (2019). Verbascoside Suppresses the Migration and Invasion of Human Glioblastoma Cells via Targeting C-Met-Mediated Epithelial-Mesenchymal Transition. Biochem. Biophys. Res. Commun..

[67] Jia W.-Q., Wang Z.-T., Zou M.-M., Lin J.-H., Li Y.-H., Zhang L., Xu R.-X. (2018). Verbascoside Inhibits Glioblastoma Cell Proliferation, Migration and Invasion While Promoting Apoptosis Through Upregulation of Protein Tyrosine Phosphatase SHP-1 and Inhibition of STAT3 Phosphorylation. Cell. Physiol. Biochem..

[68] Wang C., Wang Z., Chen C., Fu X., Wang J., Fei X., Yan X., Xu R. (2020). A Low MW Inhibitor of CD44 Dimerization for the Treatment of Glioblastoma. Br. J. Pharmacol..

[69] Wang H., Feng J., Ao F., Tang Y., Xu P., Wang M., Huang M. (2020). Tumor-Derived Exosomal microRNA-7-5p Enhanced by Verbascoside Inhibits Biological Behaviors of Glioblastoma in Vitro and in Vivo. Mol. Ther. Oncolytics.

[70] Piwowarczyk L., Mlynarczyk D.T., Krajka-Kuźniak V., Majchrzak-Celińska A., Budzianowska A., Tomczak S., Budzianowski J., Woźniak-Braszak A., Pietrzyk R., Baranowski M. (2022). Natural Compounds in Liposomal Nanoformulations of Potential Clinical Application in Glioblastoma. Cancers.

[71] Llovet J.M., Zucman-Rossi J., Pikarsky E., Sangro B., Schwartz M., Sherman M., Gores G. (2016). Hepatocellular Carcinoma. Nat. Rev. Dis. Primers.

[72] Kityania S., Nath R., Nath D., Patra J.K., Talukdar A.D. (2023). Acteoside (Verbascoside): A Prospective Therapeutic Alternative against Hepatocellular Carcinoma by Inhibiting the Expression of AXL, FGFR, BRAF, TIE2 and RAF1 Targets. Comb. Chem. High Throughput Screen..

[73] (2010). Centers for Disease Control and Prevention (CDC) Hepatocellular Carcinoma—United States, 2001–2006. MMWR Morb. Mortal. Wkly. Rep..

[74] Sim H.-W., Knox J. (2018). Hepatocellular Carcinoma in the Era of Immunotherapy. Curr. Probl. Cancer.

[75] Khullar M., Sharma A., Wani A., Sharma N., Sharma N., Chandan B.K., Kumar A., Ahmed Z. (2019). Acteoside Ameliorates Inflammatory Responses through NFκB Pathway in Alcohol Induced Hepatic Damage. Int. Immunopharmacol..

[76] Saracoglu I., Inoue M., Calis I., Ogihara Y. (1995). Studies on Constituents with Cytotoxic and Cytostatic Activity of Two Turkish Medicinal Plants Phlomis Armeniaca and Scutellaria Salviifolia. Biol. Pharm. Bull..

[77] Jiang J., Cheng R., Song A., Lou Y., Fan G. (2024). Multi-Omics Analysis Reveals Mechanism of Schisandra Chinensis Lignans and Acteoside on EMT in Hepatoma Cells via ERK1/2 Pathway. Funct. Integr. Genom..

[78] Sharma P., Prakash O., Shukla A., Rajpurohit C.S., Vasudev P.G., Luqman S., Srivastava S.K., Pant A.B., Khan F. (2016). Structure-Activity Relationship Studies on Holy Basil (*Ocimum sanctum* L.) Based Flavonoid Orientin and Its Analogue for Cytotoxic Activity in Liver Cancer Cell Line HepG2. Comb. Chem. High Throughput Screen..

[79] Tao J.-Y., Li J., Wan L., Dong B.-Z., Yu Y.-J., Liu Y.-M., Yi M.-L., Wan L.-P. (2023). Orientin Regulates the Proliferation and Migration of Hepatocellular Carcinoma Cells. Naunyn. Schmiedebergs Arch. Pharmacol..

[80] Gao L.-L., Jia D., Shi J.-Q., Zhang J.-C., Lu L.-X., Wei M., Qiao Y.-Q., Yu X.-H., Zheng Y. (2025). Acteoside Suppresses Hepatocellular Carcinoma Progression via Modulation of Macrophage Migration Inhibitory Factor and Mitogen-Activated Protein Kinase Proteins. Int. J. Biol. Macromol..

[81] Zheng Y., Jia R., Li J., Tian X., Qian Y. (2022). Curcumin- and Resveratrol-Co-Loaded Nanoparticles in Synergistic Treatment of Hepatocellular Carcinoma. J. Nanobiotechnology.

[82] Amaroli A., Panfoli I., Bozzo M., Ferrando S., Candiani S., Ravera S. (2024). The Bright Side of Curcumin: A Narrative Review of Its Therapeutic Potential in Cancer Management. Cancers.

[83] Anand P., Kunnumakkara A.B., Newman R.A., Aggarwal B.B. (2007). Bioavailability of Curcumin: Problems and Promises. Mol. Pharm..

[84] Bhattacharya S., Perris A., Jawed J.J., Hoda M. (2023). Therapeutic Role of Resveratrol against Hepatocellular Carcinoma: A Review on Its Molecular Mechanisms of Action. Pharmacol. Res.-Mod. Chin. Med..

[85] Wen L., Zhang J., Ju B., Ran Z., Zhang H., Liao Y., Cao L., Hou Q., Hu J., Yang J. (2025). Synergistic and Toxicity-reducing Effects of Acteoside as an Adjuvant Therapy of Oxaliplatin against Hepatocellular Carcinoma. Int. J. Oncol..

[86] Tsai C.-H., Kung P.-T., Kuo W.-Y., Tsai W.-C. (2020). Effect of Time Interval from Diagnosis to Treatment for Non-Small Cell Lung Cancer on Survival: A National Cohort Study in Taiwan. BMJ Open.

[87] Schabath M.B., Cote M.L. (2019). Cancer Progress and Priorities: Lung Cancer. Cancer Epidemiol. Biomark. Prev..

[88] Bade B.C., Dela Cruz C.S. (2020). Lung Cancer 2020: Epidemiology, Etiology, and Prevention. Clin. Chest Med..

[89] Jensen A.R., Mainz J., Overgaard J. (2002). Impact of Delay on Diagnosis and Treatment of Primary Lung Cancer. Acta Oncol..

[90] Chittasupho C., Athikomkulchai S., Samee W., Na Takuathung M., Yooin W., Sawangrat K., Saenjum C. (2023). Phenylethanoid Glycoside-Enriched Extract Prepared from Clerodendrum Chinense Leaf Inhibits A549 Lung Cancer Cell Migration and Apoptosis Induction through Enhancing ROS Production. Antioxidants.

[91] Khalil H.E., Ibrahim H.-I.M., Ahmed E.A., Emeka P.M., Alhaider I.A. (2022). Orientin, a Bio-Flavonoid from *Trigonella hamosa* L., Regulates COX-2/PGE-2 in A549 Cell Lines via miR-26b and miR-146a. Pharmaceuticals.

[92] Ahmed B., Qadir M.I., Ghafoor S. (2020). Malignant Melanoma: Skin Cancer-Diagnosis, Prevention, and Treatment. Crit. Rev. Eukaryot. Gene Expr..

[93] Runkle G.P., Zaloznik A.J. (1994). Malignant Melanoma. Am. Fam. Physician.

[94] Cummins D.L., Cummins J.M., Pantle H., Silverman M.A., Leonard A.L., Chanmugam A. (2006). Cutaneous Malignant Melanoma. Mayo Clin. Proc..

[95] Sandru A., Voinea S., Panaitescu E., Blidaru A. (2014). Survival Rates of Patients with Metastatic Malignant Melanoma. J. Med. Life.

[96] Wu Y., Zeng M., Xu R., Zhang B., Wang S., Li B., Kan Y., Cao B., Zheng X., Feng W. (2021). Inhibitory Activity of Acteoside in Melanoma via Regulation of the ERβ-Ras/Raf1-STAT3 Pathway. Arch. Biochem. Biophys..

[97] Son Y.-O., Lee S.-A., Kim S.-S., Jang Y.-S., Chun J.-C., Lee J.-C. (2011). Acteoside Inhibits Melanogenesis in B16F10 Cells through ERK Activation and Tyrosinase Down-Regulation. J. Pharm. Pharmacol..

[98] Nagao T., Abe F., Okabe H. (2001). Antiproliferative Constituents in the Plants 7. Leaves of Clerodendron Bungei and Leaves and Bark of C. Trichotomum. Biol. Pharm. Bull..

[99] Abe F., Nagao T., Okabe H. (2002). Antiproliferative Constituents in Plants 9. Aerial Parts of Lippia Dulcis and Lippia Canescens. Biol. Pharm. Bull..

[100] Hosen M.E., Jahan Supti S., Akash S., Rahman M.E., Faruqe M.O., Manirujjaman M., Acharjee U.K., Gaafar A.-R.Z., Ouahmane L., Sitotaw B. (2023). Mechanistic Insight of Staphylococcus Aureus Associated Skin Cancer in Humans by Santalum Album Derived Phytochemicals: An Extensive Computational and Experimental Approaches. Front. Chem..

[101] Nemkov T., D’Alessandro A., Reisz J.A. (2019). Metabolic Underpinnings of Leukemia Pathology and Treatment. Cancer Rep..

[102] Wang F., Lv H., Zhao B., Zhou L., Wang S., Luo J., Liu J., Shang P. (2019). Iron and Leukemia: New Insights for Future Treatments. J. Exp. Clin. Cancer Res..

[103] Ci T., Zhang W., Qiao Y., Li H., Zang J., Li H., Feng N., Gu Z. (2022). Delivery Strategies in Treatments of Leukemia. Chem. Soc. Rev..

[104] Collins S.J. (1987). The HL-60 Promyelocytic Leukemia Cell Line: Proliferation, Differentiation, and Cellular Oncogene Expression. Blood.

[105] Inoue M., Sakuma Z., Ogihara Y., Saracoglu I. (1998). Induction of Apoptotic Cell Death in HL-60 Cells by Acteoside, a Phenylpropanoid Glycoside. Biol. Pharm. Bull..

[106] Pettit G.R., Numata A., Takemura T., Ode R.H., Narula A.S., Schmidt J.M., Cragg G.M., Pase C.P. (1990). Antineoplastic Agents, 107. Isolation of Acteoside and Isoacteoside from Castilleja Linariaefolia. J. Nat. Prod..

[107] de Oliveira Aragão D.M., de Assis Lima I.V., da Silva J.M., Bellozi P.M.Q., de Carvalho da Costa J., Cardoso G.M.M., de Souza-Fagundes E.M., Scio E. (2013). Anti-Inflammatory, Antinociceptive and Cytotoxic Effects of the Methanol Extract of Cecropia Pachystachya Trécul. Phytother. Res..

[108] Bedell S.L., Goldstein L.S., Goldstein A.R., Goldstein A.T. (2020). Cervical Cancer Screening: Past, Present, and Future. Sex. Med. Rev..

[109] Hu Z., Ma D. (2018). The Precision Prevention and Therapy of HPV-Related Cervical Cancer: New Concepts and Clinical Implications. Cancer Med..

[110] Burd E.M. (2003). Human Papillomavirus and Cervical Cancer. Clin. Microbiol. Rev..

[111] Thaldar D.W. (2023). Who Would Own the HeLa Cell Line If the Henrietta Lacks Case Happened in Present-Day South Africa?. J. Law Biosci..

[112] Pirkkanen J.S., Boreham D.R., Mendonca M.S. (2017). The CGL1 (HeLa × Normal Skin Fibroblast) Human Hybrid Cell Line: A History of Ionizing Radiation Induced Effects on Neoplastic Transformation and Novel Future Directions in SNOLAB. Radiat. Res..

[113] Piwowarczyk L., Stawny M., Piwowarczyk K., Mlynarczyk D.T., Muszalska-Kolos I., Wierzbicka M., Goslinski T., Jelinska A. (2022). Role of Curcumin in Selected Head and Neck Lesions. Limitations on the Use of the Hep-2 Cell Line: A Critical Review. Biomed. Pharmacother..

[114] Guo Q., Tian X., Yang A., Zhou Y., Wu D., Wang Z. (2014). Orientin in Trollius Chinensis Bunge Inhibits Proliferation of HeLa Human Cervical Carcinoma Cells by Induction of Apoptosis. Monatshefte Chem. Chem. Mon..

[115] Huang F.-L., Yu S.-J. (2018). Esophageal Cancer: Risk Factors, Genetic Association, and Treatment. Asian J. Surg..

[116] Domper Arnal M.J., Ferrández Arenas Á., Lanas Arbeloa Á. (2015). Esophageal Cancer: Risk Factors, Screening and Endoscopic Treatment in Western and Eastern Countries. World J. Gastroenterol..

[117] Harada K., Rogers J.E., Iwatsuki M., Yamashita K., Baba H., Ajani J.A. (2020). Recent Advances in Treating Oesophageal Cancer. F1000Research.

[118] Zhou N., Rajaram R., Hofstetter W.L. (2020). Management of Locally Advanced Esophageal Cancer. Surg. Oncol. Clin. N. Am..

[119] Ji M., Sun J., Zhao J. (2022). Verbascoside Represses Malignant Phenotypes of Esophageal Squamous Cell Carcinoma Cells by Inhibiting CDC42 via the HMGB1/RAGE Axis. Hum. Exp. Toxicol..

[120] An F., Wang S., Tian Q., Zhu D. (2015). Effects of Orientin and Vitexin from Trollius Chinensis on the Growth and Apoptosis of Esophageal Cancer EC-109 Cells. Oncol. Lett..

[121] Mulani S.K., Guh J.-H., Mong K.-K.T. (2014). A General Synthetic Strategy and the Anti-Proliferation Properties on Prostate Cancer Cell Lines for Natural Phenylethanoid Glycosides. Org. Biomol. Chem..

[122] Wu C.-H., Chen C.-H., Hsieh P.-F., Lee Y.-H., Kuo W.W.-T., Wu R.C.-Y., Hung C.-H., Yang Y.-L., Lin V.C. (2021). Verbascoside Inhibits the Epithelial-Mesenchymal Transition of Prostate Cancer Cells through High-Mobility Group Box 1/Receptor for Advanced Glycation End-Products/TGF-β Pathway. Environ. Toxicol..

[123] Marcoccia D., Georgiev M.I., Alipieva K.I., Lorenzetti S. (2014). Inhibition of the DHT-Induced PSA Secretion by Verbascum Xanthophoeniceum and Serenoa Repens Extracts in Human LNCaP Prostate Epithelial Cells. J. Ethnopharmacol..

[124] Ji L., Yun Z., Hong Z., Baoning S., Rongliang Z. (1997). Differentiation of Human Gastric Adenocarcinoma Cell Line MGc80-3 Induced by Verbascoside. Planta Med..

[125] Ren Y., He J., Zhao W., Ma Y. (2022). The Anti-Tumor Efficacy of Verbascoside on Ovarian Cancer via Facilitating CCN1-AKT/NF-κB Pathway-Mediated M1 Macrophage Polarization. Front. Oncol..

[126] Zhang Y., Yuan Y., Wu H., Xie Z., Wu Y., Song X., Wang J., Shu W., Xu J., Liu B. (2018). Effect of Verbascoside on Apoptosis and Metastasis in Human Oral Squamous Cell Carcinoma. Int. J. Cancer.

[127] Huang Y., Wu W., Zhang X. (2024). Verbascoside Inhibits Oral Squamous Cell Carcinoma Cell Proliferation, Migration, and Invasion by the Methyltransferase 3-Mediated microRNA-31-5p/Homeodomain Interacting Protein Kinase 2 Axis. Arch. Oral Biol..

[128] Hwang Y.P., Kim H.G., Choi J.H., Park B.H., Jeong M.H., Jeong T.C., Jeong H.G. (2011). Acteoside Inhibits PMA-Induced Matrix Metalloproteinase-9 Expression via CaMK/ERK- and JNK/NF-κB-Dependent Signaling. Mol. Nutr. Food Res..

[129] Tian F., Tong M., Li Z., Huang W., Jin Y., Cao Q., Zhou X., Tong G. (2019). The Effects of Orientin on Proliferation and Apoptosis of T24 Human Bladder Carcinoma Cells Occurs Through the Inhibition of Nuclear Factor-kappaB and the Hedgehog Signaling Pathway. Med. Sci. Monit..

[130] Halim A.-B. (2020). Do We Have a Satisfactory Cell Viability Assay? Review of the Currently Commercially-Available Assays. Curr. Drug Discov. Technol..

[131] van Tonder A., Joubert A.M., Cromarty A.D. (2015). Limitations of the 3-(4,5-Dimethylthiazol-2-Yl)-2,5-Diphenyl-2H-Tetrazolium Bromide (MTT) Assay When Compared to Three Commonly Used Cell Enumeration Assays. BMC Res. Notes.

[132] Wang P., Henning S.M., Heber D. (2010). Limitations of MTT and MTS-Based Assays for Measurement of Antiproliferative Activity of Green Tea Polyphenols. PLoS ONE.

[133] Lewis M.A., Patil K., Ettayebi K., Estes M.K., Atmar R.L., Ramani S. (2024). Divergent Responses of Human Intestinal Organoid Monolayers Using Commercial in Vitro Cytotoxicity Assays. PLoS ONE.

[134] Lewandowska U., Gorlach S., Owczarek K., Hrabec E., Szewczyk K. (2014). Synergistic Interactions Between Anticancer Chemotherapeutics and Phenolic Compounds and Anticancer Synergy Between Polyphenols. Adv. Hyg. Exp. Med..

[135] Li X.-J., Zhang H.-Y. (2008). Synergy in Natural Medicines: Implications for Drug Discovery. Trends Pharmacol. Sci..

[136] Karachi A., Dastmalchi F., Mitchell D.A., Rahman M. (2018). Temozolomide for Immunomodulation in the Treatment of Glioblastoma. Neuro-Oncol..

[137] Hwang T.W., Kim D.H., Kim D.B., Jang T.W., Kim G.-H., Moon M., Yoon K.A., Choi D.E., Park J.H., Kim J.-J. (2019). Synergistic Anticancer Effect of Acteoside and Temozolomide-Based Glioblastoma Chemotherapy. Int. J. Mol. Med..

[138] Hsia T.-C., Peng S.-F., Chueh F.-S., Lu K.-W., Yang J.-L., Huang A.-C., Hsu F.-T., Wu R.S.-C. (2022). Bisdemethoxycurcumin Induces Cell Apoptosis and Inhibits Human Brain Glioblastoma GBM 8401/Luc2 Cell Xenograft Tumor in Subcutaneous Nude Mice In Vivo. Int. J. Mol. Sci..

[139] Vodenkova S., Buchler T., Cervena K., Veskrnova V., Vodicka P., Vymetalkova V. (2020). 5-Fluorouracil and Other Fluoropyrimidines in Colorectal Cancer: Past, Present and Future. Pharmacol. Ther..

[140] Tang W., Chen Z., Zhang W., Cheng Y., Zhang B., Wu F., Wang Q., Wang S., Rong D., Reiter F.P. (2020). The Mechanisms of Sorafenib Resistance in Hepatocellular Carcinoma: Theoretical Basis and Therapeutic Aspects. Signal Transduct. Target. Ther..

[141] Li N., Luo D., Hu X., Luo W., Lei G., Wang Q., Zhu T., Gu J., Lu Y., Zheng Q. (2015). RUNX2 and Osteosarcoma. Anticancer Agents Med. Chem..

[142] Jendrossek V. (2013). Targeting Apoptosis Pathways by Celecoxib in Cancer. Cancer Lett..

[143] Giordano A., Tommonaro G. (2019). Curcumin and Cancer. Nutrients.

[144] Ghosh R., Bhowmik A., Biswas S., Samanta P., Sarkar R., Pakhira S., Mondal M., Hajra S., Saha P. (2025). Natural Flavonoid Orientin Restricts 5-Fluorouracil Induced Cancer Stem Cells Mediated Angiogenesis by Regulating HIF1α and VEGFA in Colorectal Cancer. Mol. Med..

[145] Jin C., Wang K., Oppong-Gyebi A., Hu J. (2020). Application of Nanotechnology in Cancer Diagnosis and Therapy—A Mini-Review. Int. J. Med. Sci..

[146] Kuchur O.A., Tsymbal S.A., Shestovskaya M.V., Serov N.S., Dukhinova M.S., Shtil A.A. (2020). Metal-Derived Nanoparticles in Tumor Theranostics: Potential and Limitations. J. Inorg. Biochem..

[147] Burlec A.F., Corciova A., Boev M., Batir-Marin D., Mircea C., Cioanca O., Danila G., Danila M., Bucur A.F., Hancianu M. (2023). Current Overview of Metal Nanoparticles’ Synthesis, Characterization, and Biomedical Applications, with a Focus on Silver and Gold Nanoparticles. Pharmaceuticals.

[148] Hirsch L.R., Gobin A.M., Lowery A.R., Tam F., Drezek R.A., Halas N.J., West J.L. (2006). Metal Nanoshells. Ann. Biomed. Eng..

[149] Ahmadi A., Arami S. (2014). Potential Applications of Nanoshells in Biomedical Sciences. J. Drug Target..

[150] Chakraborty P., Das S.S., Dey A., Chakraborty A., Bhattacharyya C., Kandimalla R., Mukherjee B., Gopalakrishnan A.V., Singh S.K., Kant S. (2022). Quantum Dots: The Cutting-Edge Nanotheranostics in Brain Cancer Management. J. Control. Release Off. J. Control. Release Soc..

[151] Kumar S. A., Maurya D., Saikia M., Renuga Devi N., Angaiah S. (2023). A Review on Plants Derived Carbon Quantum Dots for Bio-Imaging. Mater. Adv..

[152] Akbari M., Rahimi-Nasrabadi M., Pourmasud S., Eghbali-Arani M., Banafshe H.R., Ahmadi F., Ganjali M.R., Sobhani Nasab A. (2020). CdTe Quantum Dots Prepared Using Herbal Species and Microorganisms and Their Anti-Cancer, Drug Delivery and Antibacterial Applications; a Review. Ceram. Int..

[153] Srivastav A.K., Karpathak S., Rai M.K., Kumar D., Misra D.P., Agarwal V. (2023). Lipid Based Drug Delivery Systems for Oral, Transdermal and Parenteral Delivery: Recent Strategies for Targeted Delivery Consistent with Different Clinical Application. J. Drug Deliv. Sci. Technol..

[154] Kesharwani R., Jaiswal P., Patel D.K., Yadav P.K. (2023). Lipid-Based Drug Delivery System (LBDDS): An Emerging Paradigm to Enhance Oral Bioavailability of Poorly Soluble Drugs. Biomed. Mater. Devices.

[155] Shankar R., Joshi M., Pathak K. (2018). Lipid Nanoparticles: A Novel Approach for Brain Targeting. Pharm. Nanotechnol..

[156] Li M., Du C., Guo N., Teng Y., Meng X., Sun H., Li S., Yu P., Galons H. (2019). Composition Design and Medical Application of Liposomes. Eur. J. Med. Chem..

[157] Zaimy M.A., Saffarzadeh N., Mohammadi A., Pourghadamyari H., Izadi P., Sarli A., Moghaddam L.K., Paschepari S.R., Azizi H., Torkamandi S. (2017). New Methods in the Diagnosis of Cancer and Gene Therapy of Cancer Based on Nanoparticles. Cancer Gene Ther..

[158] Gabizon A.A. (2001). Pegylated Liposomal Doxorubicin: Metamorphosis of an Old Drug into a New Form of Chemotherapy. Cancer Investig..

[159] Mukherjee A., Waters A.K., Kalyan P., Achrol A.S., Kesari S., Yenugonda V.M. (2019). Lipid-Polymer Hybrid Nanoparticles as a next-Generation Drug Delivery Platform: State of the Art, Emerging Technologies, and Perspectives. Int. J. Nanomed..

[160] Liu P., Chen G., Zhang J. (2022). A Review of Liposomes as a Drug Delivery System: Current Status of Approved Products, Regulatory Environments, and Future Perspectives. Molecules.

[161] Zhang Y., Liu B., Wu H., Li B., Xu J., Duan L., Jiang C., Zhao X., Yuan Y., Zhang G. (2014). Anti-Tumor Activity of Verbascoside Loaded Gold Nanoparticles. J. Biomed. Nanotechnol..

[162] Chen M., Zhang Y., Huang B., Yang X., Wu Y., Liu B., Yuan Y., Zhang G. (2013). Evaluation of the Antitumor Activity by Ni Nanoparticles with Verbascoside. J. Nanomater..

[163] Chittasupho C., Samee W., Na Takuathung M., Okonogi S., Nimkulrat S., Athikomkulchai S. (2024). Clerodendrum Chinense Stem Extract and Nanoparticles: Effects on Proliferation, Colony Formation, Apoptosis Induction, Cell Cycle Arrest, and Mitochondrial Membrane Potential in Human Breast Adenocarcinoma Breast Cancer Cells. Int. J. Mol. Sci..

[164] Ma Z., Zhao X., Jiang C., Yu J., Wu J., Zeng X. (2016). Gold Nanoshells with Verbascoside Induce the Apoptosis of Drug-Resistant Leukemia Cells Through Caspases Pathway and Inhibit Tumor Growth. J. Nanosci. Nanotechnol..

[165] Zhao X.-H., Yue H.-L., Li P., Zeng X., Zhang G. (2013). Evaluation of the Antitumor Activity by CdTe QDs with Verbascoside. Nano.

[166] Srećković N.Z., Nedić Z.P., Liberti D., Monti D.M., Mihailović N.R., Stanković J.S.K., Dimitrijević S., Mihailović V.B. (2021). Application Potential of Biogenically Synthesized Silver Nanoparticles Using *Lythrum salicaria* L. Extracts as Pharmaceuticals and Catalysts for Organic Pollutant Degradation. RSC Adv..

[167] Xiao Y., Ren Q., Wu L. (2022). The Pharmacokinetic Property and Pharmacological Activity of Acteoside: A Review. Biomed. Pharmacother. Biomedecine Pharmacother..

[168] Raza Ishaq A., A S El-Nashar H., M Al-Qaaneh A., Asfandyar, Bashir A., Younis T. (2025). Orientin: A Natural Glycoside with Versatile Pharmacological Activities. Nat. Prod. Res..

[169] Fahmy M.I., Sadek M.A., Abdou K., El-Dessouki A.M., El-Shiekh R.A., Khalaf S.S. (2025). Orientin: A Comprehensive Review of a Promising Bioactive Flavonoid. Inflammopharmacology.

[170] Liu S., Lyu Y., Yu S., Cheng J., Zhou J. (2021). Efficient Production of Orientin and Vitexin from Luteolin and Apigenin Using Coupled Catalysis of Glycosyltransferase and Sucrose Synthase. J. Agric. Food Chem..

[171] Rahmat E., Okello D., Kim H., Lee J., Chung Y., Komakech R., Kang Y. (2021). Scale-up Production of *Rehmannia Glutinosa* Adventitious Root Biomass in Bioreactors and Improvement of Its Acteoside Content by Elicitation. Ind. Crops Prod..

[172] Isacchi B., Bergonzi M.C., Iacopi R., Ghelardini C., Galeotti N., Bilia A.R. (2017). Liposomal Formulation to Increase Stability and Prolong Antineuropathic Activity of Verbascoside. Planta Med..

[173] Mohammed R.A., Kamel A.S., Hindam M.O., El-Dessouki A.M., Hamouda H.A., Ramadan N.M., Mohamed S.S., El-Shiekh R.A., Kamel N.M. (2025). Acteoside as a Multifunctional Natural Glycoside: Therapeutic Potential across Various Diseases. Inflammopharmacology.

[174] Dewi M.K., Chaerunisaa A.Y., Muhaimin M., Joni I.M. (2022). Improved Activity of Herbal Medicines through Nanotechnology. Nanomaterials.

